# Novel crosstalk between Vps26a and Nox4 signaling during neurogenesis

**DOI:** 10.1038/s41418-018-0226-0

**Published:** 2018-11-21

**Authors:** Seon-A Choi, Young-Hyun Kim, Young-Ho Park, Hae-Jun Yang, Pil-Soo Jeong, Jae-Jin Cha, Seung-Bin Yoon, Ji-Su Kim, Bong-Seok Song, Jong-Hee Lee, Bo-Woong Sim, Jae-Won Huh, In-Sung Song, Sang-Rae Lee, Min-Kyu Kim, Jin-Man Kim, Yun Soo Bae, Kazuhiko Imakawa, Sun-Uk Kim, Kyu-Tae Chang

**Affiliations:** 1 Futuristic Animal Resource & Research Center, Korea Research Institute of Bioscience and Biotechnology, Chungcheongbuk-do, 28116 Republic of Korea; 2 National Primate Research Center, Korea Research Institute of Bioscience and Biotechnology, Chungcheongbuk-do, 28116 Republic of Korea; 3 Laboratory of Animal Reproduction and Physiology, Department of Animal Science and Biotechnology, College of Agriculture and Life Science, Chungnam National University, Daejeon, 34134 Republic of Korea; 4 Department of Functional Genomics, University of Science and Technology, Daejeon, 34113 Republic of Korea; 5 Department of Biomedical Sciences, College of Medicine, Ulsan University, Asan Medical Center, Seoul, 05505 Republic of Korea; 6 College of Medicine, Chungnam National University, Daejeon, 34134 Republic of Korea; 7 Department of Life Science, Ewha Womans University, Seoul, 03760 Republic of Korea; 8 Animal Resource Science Center, Graduate School of Agricultural and Life Sciences, The University of Tokyo, Ibaraki, 319-0206 Japan; 9 Institute of Agricultural Sciences, Tokai University, Kumamoto, 862-8652 Japan

**Keywords:** Cell biology, Molecular biology, Cell biology, Molecular biology, Cell biology

## Abstract

Despite numerous studies on the molecular switches governing the conversion of stemness to differentiation in embryonic stem cells (ESCs), little is known about the involvement of the retromer complex. Under neural differentiation conditions, *Vps26a* deficiency (*Vps26a*^*-/-*^) or knockdown suppressed the loss of stemness and subsequent neurogenesis from ESCs or embryonic carcinoma cells, respectively, as evidenced by the long-lasting expression of stemness markers and the slow appearance of neuronal differentiation markers. Interestingly, relatively low reactive oxygen species (ROS) levels were generated during differentiation of *Vps26a*^*-/-*^ ESCs, and treatment with an antioxidant or inhibitor of NADPH oxidase (Nox), a family of ROS-generating enzymes, led to restoration of stemness in wild-type cells to the level of *Vps26a*^*-/-*^ cells during neurogenesis. Importantly, a novel interaction between Vps26a and Nox4 linked to the activation of ERK1/2 depended highly on ROS levels during neurogenesis, which were strongly suppressed in differentiating *Vps26a*^*-/-*^ ESCs. Moreover, inhibition of phosphorylated ERK1/2 (pERK1/2) resulted in decreased ROS and Nox4 levels, indicating the mutual dependency between pERK1/2 and Nox4-derived ROS during neurogenesis. These results suggest that Vps26a regulates stemness by actively cooperating with the Nox4/ROS/ERK1/2 cascade during neurogenesis. Our findings have important implications for understanding the regulation of stemness via crosstalk between the retromer molecule and redox signaling, and may contribute to the development of ESC-based therapeutic strategies for the mass production of target cells.

## Introduction

Embryonic stem cells (ESCs), which are derived from the inner cell mass of blastocyst stage embryos, are capable of unlimited proliferation and can differentiate into multiple cell lineages [1]. Their self-renewal capabilities and pluripotency are representative features of ESCs, making them a robust and suitable model to aid our understanding of developmental biology. They are also a useful resource for the development of therapeutic strategies for incurable degenerative diseases. There have been many studies of the regulation of self-renewal and specific lineage differentiation, however, relatively little is known about the underlying mechanisms. Thus, further clarification of the molecular mechanisms governing the self-renewal and differentiation processes of ESCs is needed to yield effective target cells for academic and industrial areas.

The mammalian retromer complex consists of two subcomplexes: a vacuolar protein sorting (Vps) trimer of Vps26, Vps29, and Vps35 responsible for cargo selection, and a sorting nexin (Snx) dimer of Snx1, Snx2, Snx5, and Snx6 [2], presumably responsible for tubule or vesicle formation. The retromer complex functions as a key component of the endosomal protein sorting machinery, particularly in the endosome-to-Golgi retrieval pathway [3, 4], and participates in many biological events, such as development and disease [5–7]. Indeed, the retromer complex has been found to be closely associated with pathogenic conditions such as Alzheimer’s disease through mediation of the localization of various membrane proteins, including amyloid precursor protein [8]. Furthermore, early postimplantation development in mice required the involvement of the retromer complex, as evidenced by the embryonic lethality of Snx- or Vps26a-null embryos at days 9.5 and 11.5, respectively [9, 10]. In particular, the role of Vps26a appeared to be associated with the development of the embryonic ectoderm, which gives rise to neural-lineage cells [11, 12]. Despite compelling evidence of the involvement of Vps26a during the early postimplantation period, the underlying molecular mechanism(s) remain largely unknown.

Reactive oxygen species (ROS) are created via activation of two representative systems, namely, the activation of membrane-associated NADPH oxidases (Noxs) and mitochondrial respiratory chains, and are also secondary messengers in numerous signaling pathways, as well as being molecules responsible for cellular damage [13]. Recently, Nox-generated ROS have been suggested as important regulators or determinants of stem cell behaviors, such as the maintenance of stemness and differentiation into a variety of cell types [14, 15]. As seen in the results from a variety of mammalian cell studies, increased ROS generation leads to the activation of redox signaling pathways, including mitogen-activated protein kinase (MAPK) cascades in differentiating ESCs [16, 17], which included extracellular signal-related protein kinase1/2 (ERK1/2), c-Jun NH_2_-terminnal kinase (JNK), and p38 MAPK [18, 19]. Despite numerous studies on the link between MAPKs and ESC-mediated differentiation, no relationship has been found between redox signaling and the retromer complex in mammalian systems, including ESCs.

In this study, the null mutation retromer complex molecule Vps26a (*Vps26a*^*-/-*^) inhibited neurogenesis of ESCs through potentiation of ESC stemness. Interestingly, ROS generation was severely impaired in differentiating *Vps26a*^*-/-*^ ESCs. It is important to note that Vpa26a-dependent stemness/neurogenesis transition was tightly associated with the synergistic cooperation between Nox-derived ROS and the ERK1/2 pathway. These findings increase our understanding of retromer complex- and redox signaling-associated developmental events, including the maintenance of ESC stemness.

## Results

### Vps26a-deficiency inhibits the loss of stemness and subsequent neurogenesis from ESCs

**Fig. 1:**
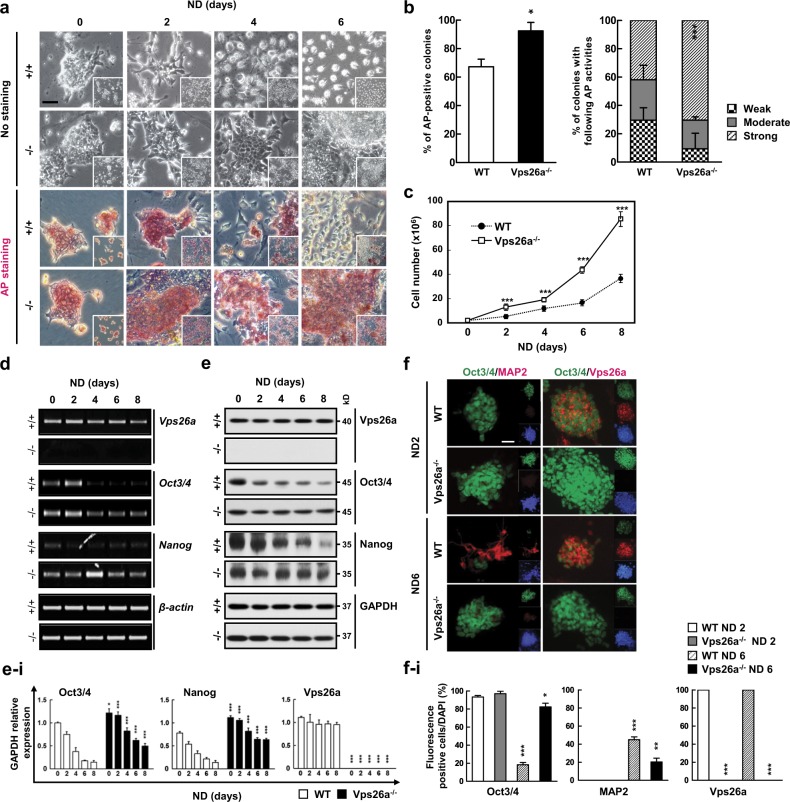
Vps26a deficiency leads to the maintenance of stemness during ESC-mediated neurogenesis. **a** Effect of Vps26a deficiency on changes in alkaline phosphatase (AP) staining during neural differentiation (ND). Wild-type (WT; +/+) and *Vps26a*^*-/-*^ (-/-) ESCs were differentiated in neurobasal medium (NBM) for the indicated time periods and subjected to AP staining. Cell clusters with differentiated morphologies are indicated by yellow dotted lines. Scale bar, 50 μm. **b** AP activities of WT and *Vps26a*^*-/-*^ ESCs differentiated for 6 days indicated by scoring of the signal intensities of at least 60 colonies from three independent experiments. **c** The number of WT and *Vps26a*^*-/-*^ cells during ND for 8 days. Error bars indicate the means ± standard deviation (SD; *n* = 3). ^***^P < 0.001 compared with WT cells each day during ND. **d**, **e** Effect of Vps26a deficiency on expression levels of ESC stemness-associated genes. +/+ and -/- ESCs cultured in NBM for the indicated days analyzed by semi-quantitative polymerase chain reaction (semi-qPCR) (**d**) and western blotting (**e**) analyses of Oct3/4 and Nanog. *β-actin* and *GAPDH* were used as loading controls. **e**–**i** Immunoblotting quantification of (**e**). Quantification of band density relative to *GAPDH* control (*n* = 3 for each group). Error bars are ± SD. ^*^P < 0.05; ^***^P < 0.001 compared with +/+ cells each day during ND. **f** Double-label immunocytochemical analysis of Oct3/4 (green) and MAP2 (red) during neural differentiation for 2 or 6 days. DAPI staining data are shown as insets to the merged images. Scale bar, 50 μm. **f**–**i** Quantitative analysis of the fluorescence. Percentage of fluorescence/DAPI-positive cells (*n* = 3 for each group). Error bars are ± SD. ^*^P < 0.05; ^**^P < 0.01; ^***^P < 0.001 vs. WT ND 2 days

To determine whether Vps26a is involved in the regulation of stemness and differentiation, ESCs were established from wild-type (WT) and *Vps26a*^*-/-*^ blastocysts at 3.5 days post coitum by expanded culture on mitotically-inactivated mouse embryonic fibroblast feeder layers (Supplementary Fig. 1), and were committed into neural-lineage cells by cultivation in neurobasal medium (NBM). Compared with the WT, colonies with an ES-like morphology and alkaline phosphatase (AP) activity were detected more frequently in differentiating *Vps26a*^*-/-*^ ESCs (Fig. 1a, b). Consistent with this, the proportion of colonies with strong AP activity was greater in *Vps26a*^*-/-*^ cells than in WT cells (Fig. 1b). Additionally, the number of cells continuously increased in the *Vps26a*^*-/-*^ group compared with WT during neural differentiation from ESCs (Fig. 1c). Semi-quantitative polymerase chain reaction (qPCR) and western blot analyses confirmed that the expression levels of the ESC stemness markers Oct3/4 and Nanog did not decrease gradually in differentiating *Vps26a*^-/-^ cells compared with WT cells (Fig. 1d–i). Similarly, cells with strong Oct3/4 immunoreactivity were frequently detected in differentiating *Vps26a*^*-/-*^ cells, whereas MAP2-postive cells were clearly stained in WT cells after 6 days of neural differentiation (Fig. 1f–i). Furthermore, HIF2α, a transcription factor of Oct3/4 and Nanog [8], was upregulated in *Vps26a*^*-/-*^ cells (Supplementary Fig. 2a and 2c). During ESC-mediated neurogenesis, no differences were found in the expression of retromer complex genes, regardless of the genotype (Supplementary Fig. 3). Vps35 knockdown influence the levels of retromer complex components including Vps26 and Vps29 [20], transfection of siVps35 decreased the *Vps26a* transcript level in P19 ECCs. Although knockdown of *Vps35* slightly affected the maintenance of *Oct3/4* in neurogenesis it did not significantly affect *MAP2* (Supplementary Fig. 4).

To determine the involvement of Vps26a in regulation of the stemness/differentiation transition, WT and *Vps26a*^*-/-*^ ESCs were proliferated anchorage-independently, leading to the formation of an embryoid body (EB), after which ESC stemness and three germ layer marker expression levels were quantified by semi-qPCR, qPCR, and western blot analyses. Under microscopic examination, a relatively small cavity size was observed in EBs compared with WT cells (Supplementary Fig. 4a). In addition, semi-qPCR and qPCR analyses revealed that transcription levels of the ESC stemness markers Oct3/4 and Nanog were maintained at higher levels in *Vps26a*^*-/-*^ EBs during an expanded culture period than in WT cells, whereas transcription levels of three germ layer markers for the ectoderm (*MAP2*, *Nestin*, and *Fgf5*), mesoderm (*Brachyury*), and endoderm (*Gata6* and *Laminin B1*) were decreased in *Vps26*^*-/-*^ EBs (Supplementary Fig. 5b and 5c). Consistent with these results, western blot analysis revealed that *Vps26a*^*-/-*^ EBs displayed an increase in the ESC stemness markers Oct3/4 and Nanog and a decrease in the neural differentiation markers MAP2 (neuron), GFAP (astrocyte), and GalC (oligodendrocyte) (Supplementary Fig. 5D). Although the size and volume of teratomas derived from *Vps26a*^*-/-*^ ESCs were significantly larger than those of WT, WT and *Vps26a*^*-/-*^ teratomas were observed in all representative derivatives of the three germ layers, including the neural rosette (ectoderm), cartilage (mesoderm), and respiratory epithelium (endoderm) (Supplementary Fig. 5a–5c). Moreover, transcription levels of the stemness markers in *Vps26a*^*-/-*^ teratomas were maintained at a higher level than those in WT teratomas, indicating that *Vps26a*^*-/-*^ ESCs exhibit differentiation resistance during teratoma formation (Supplementary Fig. 6d).

### Vps26a knockdown delayed the loss of stemness and subsequent neurogenesis in P19 embryonic carcinoma cells

**Fig. 2:**
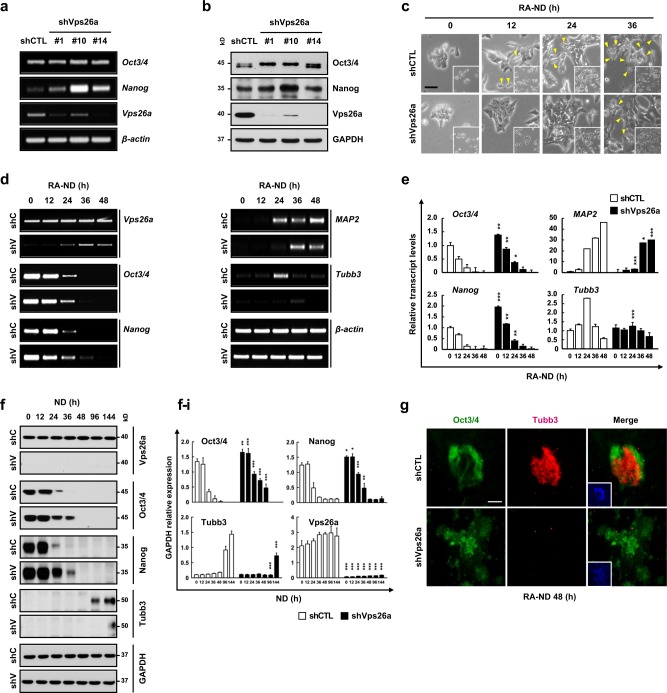
*Vps26a* knockdown prolongs the maintenance of stemness under ND conditions in P19 ECCs. **a**, **b** The effect of *Vps26a* knockdown on the expression of ESC stemness genes were determined by semi-qPCR (**a**) and western blot (**b**) analyses of Oct3/4 and Nanog using shCTL- and shVps26a-ECCs. **c** Morphological changes of shCTL (shC)- and shVps26a (shV)-ECCs during retinoic acid-induced neurogenesis (RA-ND) for the indicated time periods. The yellow arrowheads indicate cells with differentiated morphologies. **d**–**f** Expression kinetics of ESC stemness and neuronal markers examined by semi-qPCR (**d**), qPCR (**e**), and western blotting (**f**) analyses using shC- and shV-ECCs differentiated for the indicated time periods. Error bars indicate the means ± SD (*n* = 3). ^*^P < 0.05; ^**^P < 0.01; ^***^P < 0.001 compared with the shC-ECCs from each day during ND. **f**–**i** Immunoblotting quantification of (**f**). Quantification of the fluorescence intensity was performed using ImageJ software (*n* = 3 for each group). Error bars are ± SD. ^*^P < 0.05; ^**^P < 0.01; ^***^P < 0.001 vs. shCTL ND 0. **g** Double-label immunocytochemical analysis of Oct3/4 (green) and Tubb3 (red) during ND for 48 h. DAPI staining data are shown as insets to the merged images. Scale bar, 50 μm

The involvement of Vps26a in regulation of the stemness/differentiation transition was demonstrated using neurally-differentiating ESCs. For confirmation, another stem cell type, P19 mouse embryonic carcinoma cells (ECCs), stably transfected with short hairpin RNA (shRNA) targeting *Vps26a* (shVps26a-ECCs), were neurally differentiated, and the expression levels of the stemness and differentiation markers were determined. Compared with the control shRNA-transfected ECCs (shCTL-ECCs), the Vpa26a transcription and protein levels decreased to almost zero in shVps26a-ECCs (Fig. 2a, b). In contrast, Oct3/4 and Nanog were markedly upregulated in shVps26a-ECCs compared with the control. Similar to the ESCs, cell types with morphological changes were rarely detected in neurally-differentiating shVps26a-ECCs compared with the control (Fig. 2c). Semi-qPCR and qPCR analyses revealed that the transcription levels of *Oct3/4* and *Nanog* decreased gradually in differentiating shVps26a-ECCs and augmented expression of the differentiation markers *MAP2* and *Tubb3* (also known as TuJ1) was retarded (Fig. 2d, e). Consistent with these results, long-lasting expression patterns of Oct3/4 and Nanog proteins were detected mostly in differentiating shVps26a-ECCs, whereas upregulation of Tubb3 was delayed compared with the control (Fig. 2f–i). Similarly, strong immunoreactivity against Tubb3 was detected in neurally differentiated shCTL-ECCs, whereas Oct3/4-positive cells were still dominant in differentiating shVps26a-ECCs (Fig. 2g). Furthermore, Hif2α, a transcription factor of Oct3/4 and Nanog, was upregulated in *Vps26a*^*-/-*^ cells (Supplementary Fig. 2b). Consistent with the neurogenesis data, glial differentiation was also significantly reduced in differentiating *Vps26a*^*-/-*^ cells compared with the control (Supplementary Fig. 7).

### ERK1/2 participates in Vps26a-dependent regulation of the stemness/differentiation transition during neurogenesis from ESCs

**Fig. 3:**
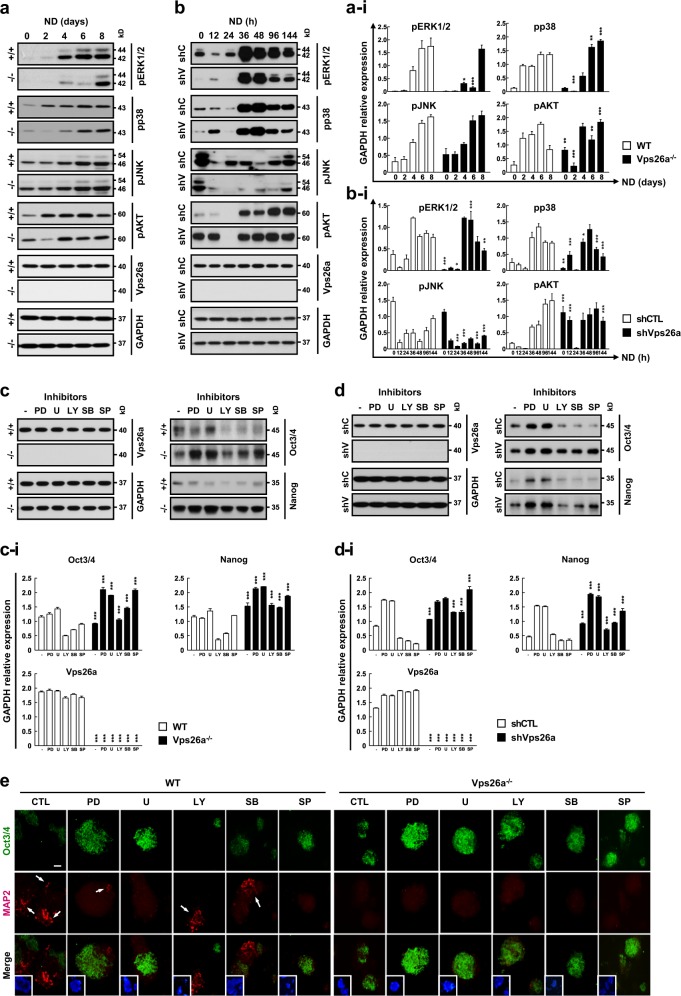
Vps26a-mediated neurogenesis depends on the ERK1/2 cascade. **a**, **b** The effects of deficiency or knockdown of Vps26a on pERK1/2, pp38 MAPK, pJNK, and pAKT levels were determined by western blotting analysis using ESCs (**a**) or ECCs (**b**), respectively, differentiated for the indicated time periods. **a**-**i**, **b**-**i** Immunoblotting quantification of (**a**) and (**b**). Western blot signals were quantified and the intensity of phosphorylated protein and the total protein normalized to the loading control GAPDH are presented (*n* = 3 for each group). Error bars are ± SD. ^*^P < 0.05; ^**^P < 0.01; ^***^P < 0.001 vs. +/+ ND 0 (**a**-**i**) and shCTL ND 0 (**b**-**i**). **c**, **d** The effect of signaling inhibitors for ERK1/2 (PD98059, PD; U0126, U), p38 MAPK (SB203580, SB), JNK (SP600125, SP), and AKT (LY294002, LY) on expression of ESC stemness markers was examined by western blot analysis of Oct3/4 and Nanog using WT (+/+) and *Vps26a*^-/-^ (-/-) ESCs differentiated for 6 days (**c**) and shCTL (shC)- and shVps26a (shV)-ECCs differentiated for 144 h (**d**). **c**-**i**, **d**-**i** Immunoblotting quantification of (**c**) and (**d**). Western blot signals were quantified and the intensity of phosphorylated protein and the total protein normalized to the loading control GAPDH are presented (*n* = 3 for each group). Error bars are ± SD. ^***^P < 0.001 vs. +/+ ND 6 (**c-i**) and shCTL ND 6 (**d**-**i**). **e** Double-label immunocytochemical analysis of Oct3/4 (green) and MAP2 (red) using WT and *Vps26a*^-/-^ ESCs differentiated in the presence or absence of the indicated signaling inhibitors for 6 days. DAPI staining data are shown as insets to the merged images. Scale bar, 50 μm

Previous studies have shown that phosphoinositide 3-kinase (PI3K) and MAPKs are involved in ESC differentiation processes [18]. Therefore, to investigate the signaling pathways governing neurogenesis, WT and *Vps26a*^*-/-*^ ESCs were differentiated in NBM and subjected to western blot analysis of phosphorylated ERK1/2 (pERK1/2), pp38 MAPK, pJNK, and pAKT. Of the kinases investigated, the most notable change in phosphorylation was detected in the pERK1/2 immunoblot during neural differentiation from ESCs (Fig. 3a–i). The gradual increase in pERK1/2 was markedly reduced in differentiating *Vps26a*^*-/-*^ ESCs compared with WT cells (Fig. 3a–i). A similar pattern of pERK1/2 expression was also present upon comparison of shCTL- and shVps26a-ECCs (Fig. 3b–i). Consistent with these results, the overexpression of Vps26a in human embryonic kidney (HEK) 293 cells increased pERK1/2 levels in response to epidermal growth factor (Supplementary Fig. 8). To examine the suppressive effects of redox-sensitive signaling pathways on the maintenance of stemness, *Vps26a*^*-/-*^ ESCs and shVps26a-ECCs were differentiated in the presence or absence of three MAPK and AKT signaling inhibitors. Next, Oct3/4 and Nanog expression levels were examined by western blot analysis. Interestingly, PD98059 and U0126, potent ERK1/2 inhibitors, upregulated Oct3/4 and Nanog levels in normal, *Vps26a-*knockout, or *Vps26a*-knockdown cases, except for Nanog levels in WT cells (Fig. 3c, d). Consistent with these results, immunocytochemistry analysis revealed restoration of Oct3/4 immunoreactivity and a decrease in MAP2-positive cells in WT cells treated with ERK1/2 inhibitors (Fig. 3e). Similarly, Oct3/4 immunoreactivity was markedly increased following treatment with ERK1/2 inhibitors in differentiating *Vps26a*^*-/-*^ ESCs (Fig. 3e).

### ROS are involved in Vps26a-mediated regulation of the stemness/differentiation transition, possibly by activating Nox4 during neurogenesis from ESCs

**Fig. 4:**
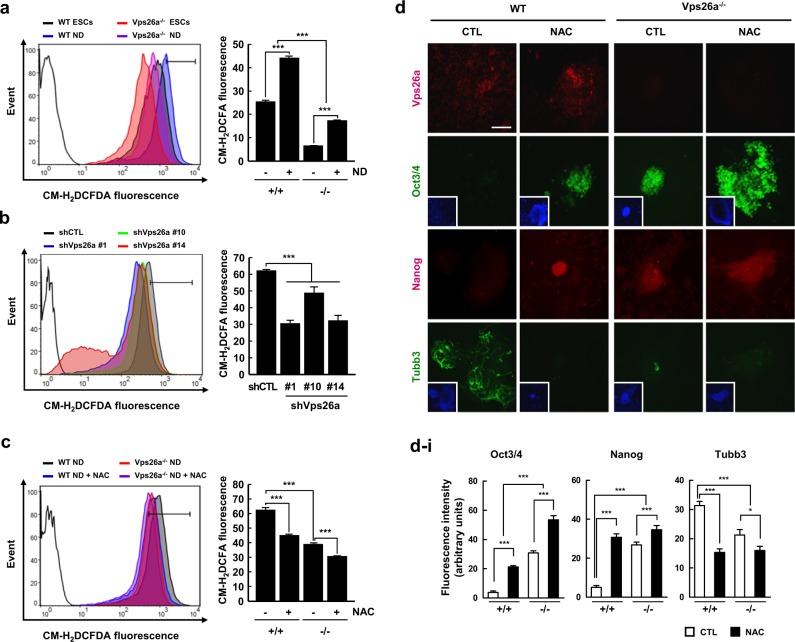
Vps26a is required for increased ROS leading to ESC-mediated neurogenesis. **a**, **b** The effect of Vps26a deficiency (**a**) or knockdown (**b**) on ROS generation was determined by flow cytometry using WT and *Vps26a*^*-/-*^ ESCs (**a**) and shCTL- and shVps26a-ECCs (#1, #10, and #14) (**b**) differentiated for 6 days and 48 h, respectively. Unstained cells (black lines) were used as a negative control. The data are representative of at least three independent experiments and presented as means ± SD (*n* = 3). ^***^P < 0.001. **c** WT and *Vps26a*^*-/-*^ ESCs were differentiated in the presence or absence of 2.5 mM NAC for 6 days, and ROS levels were measured by flow cytometry. The data are representative of at least three independent experiments and presented as means ± SD (*n* = 3). ^***^P < 0.001. **d** Double-label immunocytochemical analysis of Vps26a (red), Oct3/4 (green), Nanog (red), and Tubb3 (green) using WT and *Vps26a*^*-/-*^ ESCs differentiated in the presence or absence of 2.5 mM NAC for 3 days. DAPI staining data are shown as insets to the Oct3/4 and Tubb3 images. Scale bar, 50 μm. **d**–**i** immunofluorescence quantification of (**d**). Quantitative analysis of the fluorescence intensity performed using ImageJ software (*n* = 3). Error bars are ± SD. ^*^P < 0.05; ^***^P < 0.001 vs. WT ND 6 CTL

Evidence suggests a close association between Vps26a and several pathological conditions, including degenerative neurological diseases [12, 21]. Additionally, excessive ROS are often viewed as the hallmark of a variety of degenerative diseases, with the redox status acting as a central modulator of the stemness/differentiation transition in differentiating ESCs [19]. Furthermore, numerous reports have demonstrated that ROS are involved in the activation of MAPK cascades [16, 17]. Based on these findings, ROS levels were measured in undifferentiated and differentiating ESCs stained with CM-H_2_DCFDA, an indicator of ROS, by flow cytometry. ROS levels were elevated during neural differentiation from ESCs (Fig. 4a). Interestingly, *Vps26a* deficiency caused a decrease in ROS levels in undifferentiated and differentiating ESCs compared with WT cells (Fig. 4a), as evidenced by flow cytometric analysis using shCTL- and shVps26a-ECCs (Fig. 4b). Moreover, the reduction in ROS following treatment with N-acetyl L-cysteine (NAC), a chemical ROS scavenger, restored the immunoreactivity of Oct3/4 and Nanog in differentiating WT ESCs to the levels of *Vps26a*^*-/-*^ cells (Fig. 4c–i).

**Fig. 5:**
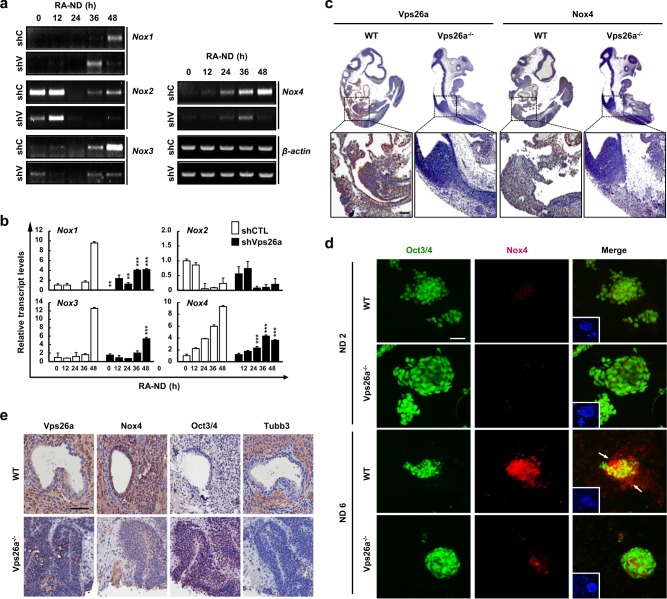
Vps26a involves Nox expression during neurogenesis. **a**, **b** The effect of Vps26a deficiency and knockdown on the expression of the *Nox* family was determined by semi-qPCR (**a**) and qPCR (**b**) analyses using shCTL (shC)- and shVps26a (shV)-ECCs, differentiated for the indicated time periods. Error bars indicate the means ± SD (*n* = 3). ^**^P < 0.01; ^***^P < 0.001 compared with shCTL-ECCs each day. **c** Overview of Vps26a and Nox4 immunostaining of a sagittal section of WT and *Vps26a*^*-/-*^ mouse embryos (E10.5) counterstained lightly with hematoxylin. Scale bar , 200 µm. **d** Double-label immunocytochemical analysis of Oct3/4 and Nox4 using WT and *Vps26a*^*-/-*^ ESCs differentiated for 6 days. DAPI staining data are shown as insets to the merged images. Scale bar, 50 μm. **e** Expression of Nox4, Oct3/4, and Nox4 in WT and *Vps26a*^*-/-*^ teratomas. Scale bar, 100 μm

To identify the source of ROS during neural differentiation from ESCs, the expression levels of Nox family proteins, which are representative cellular ROS-generating enzymes, were investigated by semi-qPCR and qPCR analyses using differentiating shCTL ECCs. Except for *Nox2*, most of the Nox enzymes increased during neural differentiation from ECCs (Fig. 5a, b). In particular, gradual upregulation of Nox3 and Nox4 was significantly reduced by knockdown of Vps26a in shCTL ECCs (Fig. 5a, b). Consistent with this observation, whole-mount immunohistochemistry revealed that Nox4 expression was also markedly diminished in *Vps26a*^*-/-*^ embryos compared with WT embryos at embryonic day 10.5 (E10.5) (Fig. 5c). Moreover, immunocytochemistry revealed strong Nox4 immunoreactivity in differentiating ESCs, which was greatly reduced in *Vps26a*^*-/-*^ cells compared with WT cells (Fig. 5d). Furthermore, *Vps26a*^*-/-*^ ESC-derived teratomas suppressed differentiation and sustained Oct3/4 expression levels, whereas WT ESC-derived teratomas upregulated the expression of Tubb3 and Nox4 (Fig. 5e). We found that overexpression of Nox4 increased MAP2-positive cells from shVps26a ECCs, while decreased in Oct3/4-positive cells. These results demonstrate that neurogenesis capacity can be restored by regulating the gene of Nox4, which is reduced to *Vps26a* deficiency (Supplementary Fig. 9).

### Vps26a interacts with Nox4

**Fig. 6:**
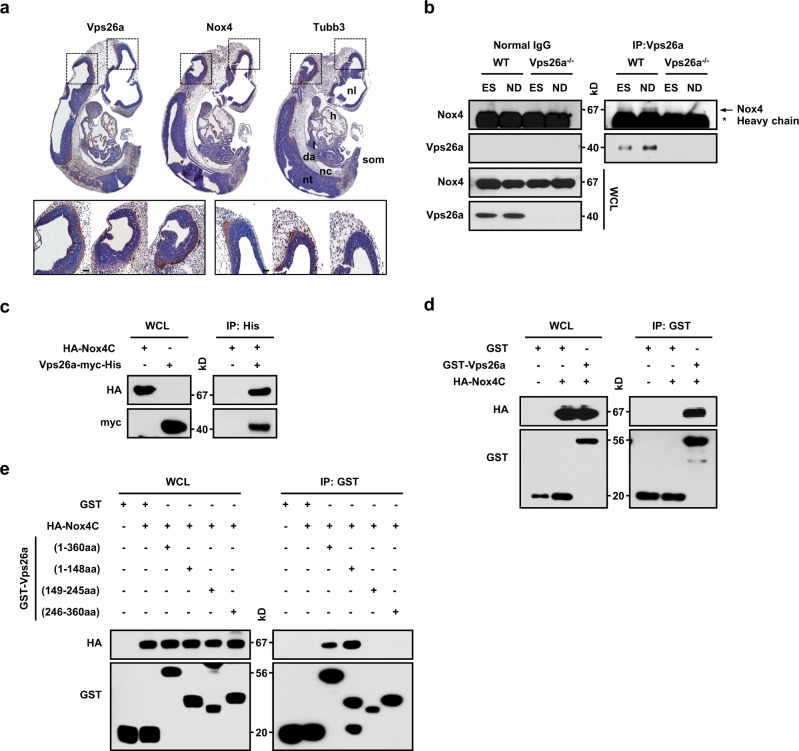
Identification of the interaction between Vps26a and Nox4. **a** Serial sagittal sections of WT embryos were immunostained for Vps26a, Nox4, and Tubb3 and counterstained lightly with hematoxylin at E10.5. da, dorsal aorta; l, lung; h, heart; nc, notochord; nl, neural lumen; nt, neural tube; som, somite. **b** Vps26a immunoprecipitation followed by anti-Vps26a and -Nox4 immunoblots using lysates obtained from WT (+/+) and *Vps26a*^*-/-*^ (-/-) ESCs during neural differentiation for 0 or 6 days. IgG was used as an immunoprecipitation control. **c** GST-tagged Vps26a was subjected to a pull-down assay with the lysates of HEK293 cells transfected with HA-Nox4C (C-terminal region, 249–574 aa)-expressing plasmid. Immunoblot analysis with anti-HA antibody is shown at the top. Equal loading of the GST proteins assessed by GST antibody is shown at the bottom. GST was used as a negative control. **d** Cell lysates from HEK293 cells transfected with HA-Nox4C were mixed with purified His-tagged Vps26a. Samples were immunoblotted with the HA tag antibody. Levels of input protein are shown by immunoblotting with the HA- and Myc-tag antibodies. **e** Expression constructs for GST-tagged serial deletion mutants of Vps26a and HA-Nox4C were co-expressed in HEK293 cells. Immunoblot analysis with anti-HA antibody is shown at the top. Equal loading of the GST proteins assessed by GST antibody is shown at the bottom. GST was used as a negative control

To identify a possible interaction between Vps26a and Nox4, we first examined whether both proteins colocalize in embryos. Most Vps26a-positive areas were precisely colocalized with areas of Nox4 in Tubb3-positive neuronal cells at E10.5 by whole-mount immunohistochemistry (Fig. 6a). Moreover, Vps26a and Nox4 exhibited a specific interaction in differentiating WT cells but not in *Vps26a*^*-/-*^ cells, as revealed by immunoprecipitation experiments in undifferentiated and differentiating ESCs, and displayed a stronger interaction, along with neuronal differentiation, accompanied by increased protein levels of Nox4 and Vps26a (Fig. 6b). Consistent with these results, HA-Nox4C coprecipitated with purified His-tagged Vps26a (Fig. 6c) and glutathione S-transferase (GST)-Vps26a (Fig. 6d) in the binding assay. To determine the region of Vps26a that interacts with Nox4, a series of Vps26a-truncated proteins tagged with GST were expressed, and their abilities to interact with HA-Nox4C were tested using a GST-pull-down assay. Nox4 interacted with the N-terminal Vps26a (amino acids; aa 1–148) protein (Fig. 6e, lane 4), whereas Nox4 failed to interact with the C-terminal Vps26a (aa 149–245 and 246–360) protein (Fig. 6e, lanes 5 and 6).

### Vps26a-mediated regulation of the stemness/differentiation transition depends on the synergy between Nox, ROS, and ERK1/2

**Fig. 7:**
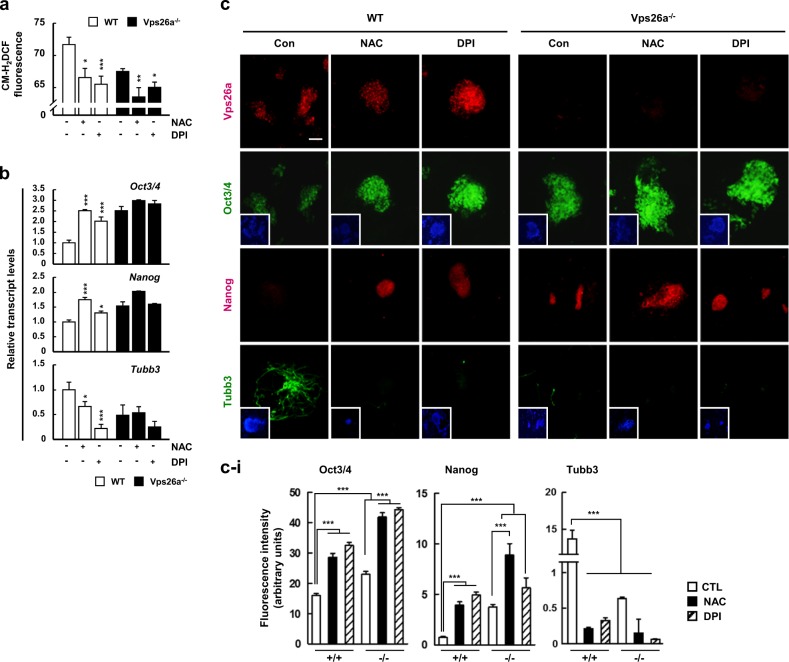
Nox4-generated ROS lead to a loss of stemness and subsequent neurogenesis from ESCs. **a**, **b** The effects of antioxidant and Nox inhibitor treatments on ROS generation (**a**) and ESC stemness transcription levels (**b**) were examined by flow cytometry using WT and *Vps26a*^*-/-*^ ESCs differentiated in the presence or absence of 2.5 mM NAC or 10 μM DPI for 6 days. Error bars indicate the means ± SD (*n* = 3). ^*^P < 0.05; ^**^P < 0.01; ^***^P < 0.001 compared with no treatment. **c** Double-label immunocytochemical analysis of Vps26a (red), Oct3/4 (green), Nanog (red), and Tubb3 (green) using WT and *Vps26a*^*-/-*^ ESCs differentiated in the presence or absence of 20 mM NAC or 2.5 μM DPI for 6 days. DAPI staining data are shown as insets to the Oct3/4 and Tubb3 images. Scale bar, 50 μm. **c-i** Immunofluorescence quantification of **c**. Quantification of the fluorescence intensity was performed using ImageJ software (*n* = 3). Error bars are ± SD. ^*^P < 0.05; ^***^P < 0.001 vs. WT ND 6 CTL

To define the involvement of Nox-generated ROS in the Vps26a-dependent transition of stemness/differentiation during neurogenesis, WT and *Vps26a*^*-/-*^ ESCs were differentiated in the presence or absence of NAC or diphenyleneiodonium (DPI), a potent Nox inhibitor, and flow cytometry was performed to measure ROS, along with qPCR and immunocytochemistry to investigate the expression of stemness/differentiation markers. Similar to treatment with the antioxidant NAC, supplementation of DPI successfully decreased ROS generation in undifferentiated and differentiating ESCs, regardless of the *Vps26a* genotype (Fig. 7a). Under reduced ROS following treatment with either NAC or DPI, *Oct3/4* and *Nanog* transcription levels in WT cells were restored to the levels of *Vps26a*^*-/-*^ ESCs, whereas the increase in *Tubb3* expression was significantly reduced in WT cells (Fig. 7b). Consistent with these findings, Oct3/4 and Nanog immunoreactivity was enhanced by treatment with either NAC or DPI, with intensities comparable with those of *Vps26a*^*-/-*^ ESCs (Fig. 7c–i). When Nox4 was reduced using siRNA, Oct3/4 and Nanog were expressed higher than siCTL, and MAP2 was remarkably decreased in ECCs with Nox4 siRNA. These results are consistent with previous findings that increased Nox4-mediated ROS production improves neural progenitor cell differentiation [22, 23] (Supplementary Fig. 10). Taken together, Nox4 seems to play an important role in neuronal differentiation through interaction with Vps26a.

**Fig. 8:**
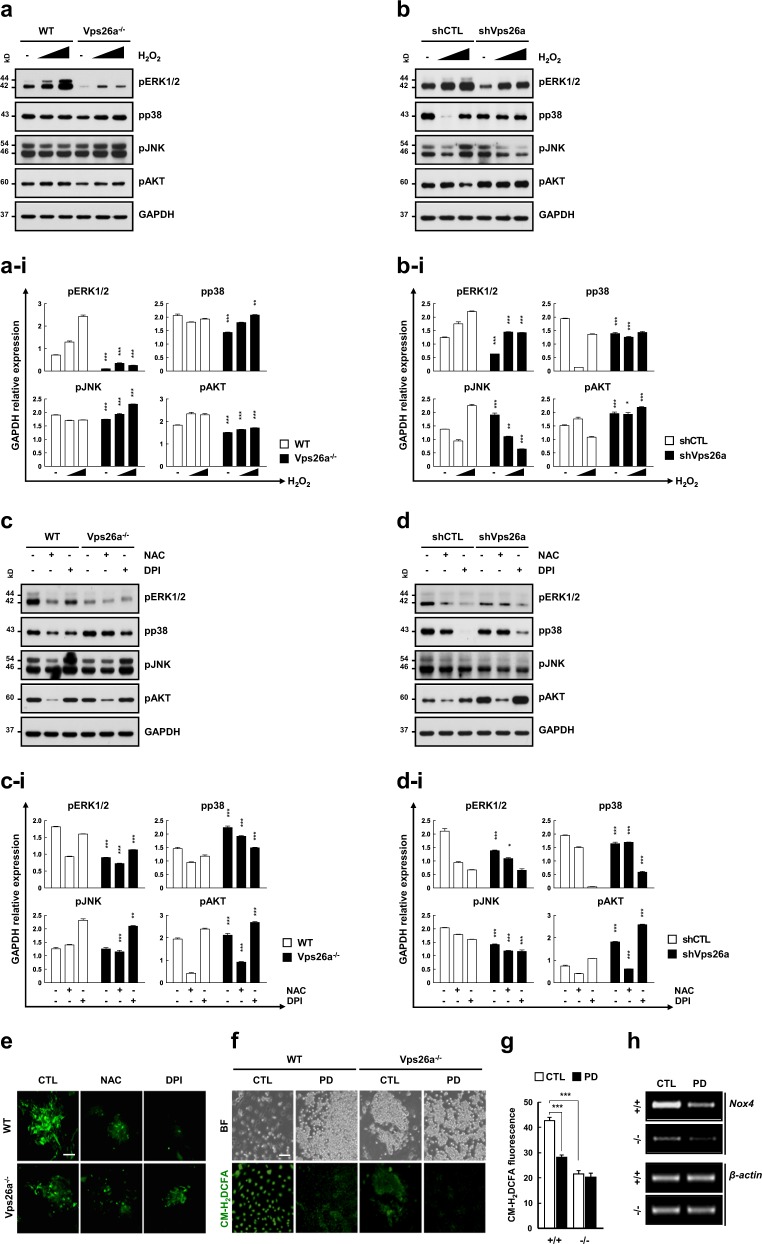
Vps26a-mediated neurogenesis depends on cooperation between Nox, ROS, and the ERK1/2 cascade. **a, b** The effects of oxidant treatment on modulation of pERK1/2, pp38 MAPK, pJNK, and pAKT levels were determined by western blot analysis using WT (+/+) and *Vps26a*^*-/-*^ (-/-) ESCs or shCTL (shC)- and shVps26a (shV)-ECCs (**b**) treated with increasing concentrations of hydrogen peroxide (50 and 100 μM), respectively, for 10 min at the end of neural differentiation (6 days). **a**–**i**, **b**–**i** Immunoblotting quantification of (**a**) and (**b**). Western blot signals were quantified and the intensity of phosphorylated protein and the total protein normalized to the loading control GAPDH are presented (*n* = 3 for each group). Error bars are ± SD. ^**^P < 0.01; ^***^P < 0.001 vs. +/+ ND 6 (**a**–**i**) and shCTL ND 6 (**b**–**i**). **c**, **d** The effects of antioxidant- and Nox inhibitor treatments on modulation of pERK1/2, pp38 MAPK, pJNK, and pAKT levels were determined by western blot analysis using WT and *Vps26a*^*-/-*^ ESCs (**c**) or shCTL- and shVps26a-ECCs (**d**) cultured in the presence or absence of 20 mM NAC or 2.5 μM DPI for 6 h at the end of neural differentiation (6 days and 144 h, respectively). **c**–**i**, **d**–**i** Immunoblotting quantification of (**c**) and (**d**). Western blot signals were quantified and the intensity of phosphorylated protein and the total protein normalized to the loading control GAPDH are presented (*n* = 3 for each group). Error bars are ± SD. ^***^P < 0.001 vs. +/+ ND 6 (**c**–**i**) and shCTL ND 6 (**d**–**i**). **e** Immunocytochemical analysis of pERK1/2 using WT and *Vps26a*^*-/-*^ ESCs differentiated in the presence or absence of 20 mM NAC or 2.5 μM DPI for 6 days. Scale bar, 50 μm. **f**, **g** The effect of ERK1/2 inhibitor treatment on ROS generation examined by fluorescence microscopy (**f**) or flow cytometry (**g**) using +/+ and -/- ESCs differentiated in the presence or absence of PD98059 for 3 days. Error bars indicate the means ± SD (*n* = 3). ^***^P < 0.001. **h** The effect of ERK inhibitor treatment on the expression of Nox4 determined by semi-qPCR analysis using +/+ and -/- ESCs differentiated in the presence or absence of PD98059 for 6 days

To determine whether ERK1/2 activation is required for Nox-generated ROS, western blot analysis of three MAPKs and AKT was performed using *Vps26a*^*-/-*^ ESCs and shVps26a-ECCs cultured or differentiated in the presence or absence of hydrogen peroxide, NAC, or DPI. Of the kinases investigated, pERK1/2 levels were markedly reduced in *Vps26a*^*-/-*^ ESCs compared with WT cells, although the levels responded to hydrogen peroxide in both genotypes (Fig. 8a, b). Additionally, treatment with NAC or DPI greatly decreased the intensity of the pERK1/2 and in WT cells, comparable with the levels in the *Vps26a*^*-/-*^ groups (Fig. 8c), showing a similar pattern to the western blot analysis using shCon- and shVps26a-ECCs (Fig. 8d). pERK1/2 immunoreactivity was markedly reduced by supplementation with either NAC or DPI during neural differentiation of WT cells (Fig. 8e), as demonstrated in the western blot analysis. More interestingly, the levels of ROS (Fig. 8f, g) and *Nox4* (Fig. 8h) were markedly reduced following treatment with PD98059, indicating crosstalk feedback between Nox4/ROS and ERK1/2 during neurogenesis from ESCs.

## Discussion

The insertion mutation of Vps26a in mice leads to embryonic lethality due to failure in early postimplantation development [10]. To date, few studies have investigated the mechanism(s) linking developmental abnormalities in *Vps26a*^*-/-*^ fetuses. To address this, *Vps26a*^*-/-*^ ESCs were generated and used as an in vitro model of differentiation. Neurogenesis analyses of these cell lines revealed that Vps26a actively participates in regulation of the stemness/differentiation transition via cooperation with redox signaling. Furthermore, these findings represent the first demonstration of an association between the retromer complex and ROS.

Researchers have investigated redox-sensitive mechanisms regulating the behavior of ESCs during differentiation, including the involvement of antioxidant enzymes. However, the expression kinetics of antioxidant enzymes does not always correlate with intracellular ROS levels. Following application of neural differentiation stimuli, the antioxidant enzymes Prx I and Prx II showed opposing expression levels due to the intercellular distribution of Prx I and Prx II in ESCs and neural-lineage cells, respectively [19]. Consistent with this finding, the present study also identified opposing expression kinetics of Prx I and Prx II during neurogenesis from ESCs, which were severely retarded in differentiating *Vps26a*^*-/-*^ ESCs compared with WT cells (Supplementary Fig. 11). These results suggest that neurogenesis from ESCs requires the stepwise and cell-specific involvement of antioxidants without greatly affecting total intracellular ROS levels. The current aim is to define the roles of specific antioxidant enzymes in their preferentially expressed cells during differentiation.

The retromer complex has been implicated in degenerative neurological diseases, including Alzheimer’s disease (AD) and Parkinson’s disease (PD) [7, 24, 25]. Null mutations in Vps26 and Vps35 in mice accelerate the production of β-amyloid (Aβ), a cleaved product of the amyloid precursor protein (APP) associated with AD pathogenesis [12] by increasing the resident time of APP within a given membrane compartment and enhancing APP β-cleavage by beta-secretase [26]. Consistent with these results, knockdown of Vps26 and Vps35 increased Aβ40 expression and secretion in HeLa- [27] and APP-overexpressing HEK293 cells [28], respectively. Recently, researchers reported retromer stabilization has a potentially beneficial effect on Aβ generation from human stem cell-derived neurons [29, 30]. Furthermore, recent studies have proposed that Vps35 mutations may cause PD [6, 31], which is evidenced by alterations in autophagic activity; [32] this is indicative of a close association between the retromer complex and neurodegenerative pathogenesis. In general, neurodegenerative disorders frequently entail oxidative stress or excessive ROS levels. ROS overloads are frequently detected in AD and PD models of cells or animals [33, 34], and altered ROS levels are generally accepted as a marker of neurological disease [35]. Nevertheless, the association between the retromer complex and redox status has not yet been clarified. Our findings identified a relationship between Vps26a and ROS that could have a marked impact on retromer complex studies in relation to incurable degenerative disorders. We aim to clarify the molecular mechanism(s) underlying the involvement of ROS in regulating the retromer complex.

Many studies have demonstrated that MAPKs, such as ERK1/2, p38 MAPK, and JNK play an important role in differentiation processes [18]. Among the three MAPKs, ERK1/2 and JNK are mostly responsible for the neural induction of ESCs [19, 36, 37]. Indeed, suppression of the ERK1/2 pathways enhanced the self-renewal of mouse ESCs [38] and retinoic acid-mediated neurogenesis required activation of ERK1/2 [37, 39]. In addition, activation of ERK1/2 by basic fibroblast growth factors contributed to neural growth and differentiation of mouse and human ESCs. In antioxidant enzyme *Prx I*^*-/-*^ or *Prx II*^*-/-*^ cells, inhibition of JNK, but not ERK1/2, signaling provided the greatest protection against ROS-mediated loss of stemness, demonstrating that hyperactivation of JNK could override the action of ERK1/2 during neurogenesis under conditions of excessive ROS [19]. In this study, the role of Vps26a in neurogenesis mainly depended on ERK1/2 rather than JNK. Failure to activate the ERK1/2 cascade in differentiating *Vps26a*^*-/-*^ ESCs resulted in prolonged maintenance of stemness and delayed neurogenesis (Fig. 3a, b), which was further evidenced by the mutual dependency between the Nox/ROS and ERK1/2 cascades during neurogenesis from ESCs. Thus, it was presumed that ERK1/2 functions as a central regulator of the stemness/differentiation transition via cooperation with the Nox/ROS cascade.

Nox also participates in the differentiation process of various cell types as a modulator of intracellular ROS signaling. Recent studies have shown that the redox status is closely associated with the commitment of stem cells to cardiac myocytes [40]. In addition, Nox4 activation is required for the differentiation of neonatal c-kit^+^ cardiac precursor cells to smooth muscle cells [41]. Furthermore, BMP2 elevated ROS levels in neural crest stem cells (NCSCs), and treatment with a Nox inhibitor suppressed neuronal differentiation from NCSCs [22]. In particular, knockdown of Nox4 appeared to be critical for the survival of NCSCs (at least in vitro), and *Nox4*^*-/-*^ mouse embryos showed delayed overall development, indicating that Nox4-mediated ROS generation likely plays an important role in the differentiation of NCSCs during embryogenesis [22]. Our data are consistent with other reports, which describe retinoic acid (RA)-induced cell differentiation is characterized by the increase in Nox4 mRNA and protein expression [23, 42], and Nox4-generated ROS stimulatory effects on neural stem cell proliferation [43, 44]. Moreover, a role for ERK1/2 signaling pathway by retinoic acid that is required for neural differentiation has been already shown in ESCs [45, 46], and we confirmed that RA-induced upregulation of Nox4 promotes the differentiation of ESCs into neural cells-activated MAPK kinase. The present study revealed that the defects of differentiating *Vps26a*^*–/*–^ ESCs may be closely related to reduced Nox4 levels. Experiments using a Nox inhibitor revealed that Vps26a-mediated neurogenesis from ESCs required the activation of Nox4. Interestingly, Nox4 and ERK1/2 activation were mutually dependent for successful neurogenesis, which is in good accordance with the results obtained from induction of neuronal differentiation by H_2_O_2_ concentration that was induced more in agreement with increased pERK1/2 (Supplementary Fig. 12). These results strongly suggest that Vps26a-mediated neurogenesis greatly depends on synergistic cooperation between Nox4, ROS, and ERK1/2.

We aimed to provide compelling evidence of a relationship between the retromer complex and ESC behavior, which led to the proposition of a comprehensive model and identification of the mechanism by which the stemness/differentiation transition is regulated via cooperation between Vps26a and redox signaling (Supplementary Fig. 13). In particular, activation of the Nox4/ROS/ERK1/2 pathway was severely impaired in differentiating *Vps26a*^*-/-*^ cells, suppressing ESC-mediated neurogenesis. These findings represent new evidence of an association between Vps26a and redox signaling pathways, and may contribute to the development of alternative stem cell-based therapeutic strategies for the mass production of high-quality neurons.

## Materials and methods

### Cell culture

ESCs were grown in Dulbecco’s modified Eagle’s medium (DMEM; Invitrogen) containing 5% ES-qualified fetal bovine serum (FBS; Invitrogen), 10% FBS (Hyclone), 1× non-essential amino acids (NEAA; Invitrogen), 500 U/ml leukemia inhibitory factor (LIF; Chemicon, Temecula, CA, USA), and antibiotics (Invitrogen) on mitotically-inactivated mouse embryonic fibroblasts (MIMEFs). The culture medium for P19 mouse embryonic carcinoma cells (ECCs) consisted of DMEM containing 10% FBS (GIBCO) and antibiotics.

### Alkaline phosphatase activity assay

All activity assay procedures were performed according to the manufacturer’s instructions (Sigma-Aldrich, Leukocyte Alkaline Phosphatase Kit, Cat# 86R). Briefly, after the culture medium had been discarded, the cells were fixed with citrate–acetone–formaldehyde fixative for 2 min at room temperature, and rinsed with distilled water. They were then incubated with 200 μg/ml Naphthol AS-MX phosphate (Sigma-Aldrich) and 1 mg/ml Fast Red TR salt (Sigma-Aldrich) in 100 mM Tris buffer at pH 8.2 for 15 min at room temperature. The staining reaction was terminated by rinsing with PBS.

### Embryoid body (EB) formation

An ESC suspension (10 ml) with a density of 10^3^–10^6^ cells/ml was seeded into a 100-mm bacterial-grade dish. Seeded ESCs did not attach to the plastic surfaces of the bacterial-grade dishes, and naturally stuck to each other to form aggregates, without shaking [47]. The EB sizes were measured from collected micrographs using ImageJ software (developed at the U.S. National Institutes of Health and available at http://rsb.info.nih.gov/nih-image/).

### Semi-qPCR and real-time RT-qPCR

Total RNAs were extracted from cells using the RNeasy plus mini kit (Qiagen) according to the manufacturer’s instructions. Total RNA (1 μg) was used for cDNA synthesis using ReverTra Ace-α^®^ (Toyobo). The expression levels of each gene were determined by semi-qPCR methods. The PCR was carried out in a 20 μl reaction volume using the ExPrime Taq Master Mix (Genetbio). Each cycle consisted of a denaturation step at 94 °C for 20 s, an annealing step at 62 °C for 20 s, and an extension step at 72 °C for 20 s. The final extension step was followed by a 5 min extension reaction at 72 °C. Relative expression levels of the genes were measured by real-time RT-PCR using Brilliant III Ultra-Fast SYBR^®^ Green QPCR Master Mix (Stratagene) and analyzed with an Mx3000P QPCR systems (Stratagene). For the comparative analyses, mRNA expression levels were normalized to GAPDH and then expressed as the fold change. The sample delta Ct (SΔCt) value was calculated from the differences between the Ct values of GAPDH and the target genes. The relative gene expression levels between the samples and the controls were determined using the formula 2−(SΔCt−CΔCt). PCR primers for amplification of the human and mouse cDNAs were designed in silico using Primer3 software (http://frodo.wi.mit.edu/primer3/input.htm; Supplementary information, Table S1).

### Western blot analysis

For the western blot analysis, 30−60 μg of protein lysates were separated in 8−12% sodium dodecyl sulfate-polyacrylamide gels and transferred onto nitrocellulose membranes (Millipore). The membranes were incubated overnight with primary antibodies (Supplementary Information, Table S2) at 4 °C. The membranes were then washed five times with 10 mM Tris–HCl (pH 7.5) containing 150 mM NaCl and 0.2% Tween-20 (TBST) and incubated with horseradish peroxidase-conjugated goat anti-rabbit IgG or anti-mouse IgG (1:5000, both from Sigma-Aldrich) for 1 h at room temperature. After the blots were washed with TBST, antibody binding was detected using a chemiluminescence detection system (Amersham) according to the manufacturer’s instructions. Western blot band density was quantified with ImageJ software. Representative mean values were of at least three independent experiments with standard deviation.

### Double-label immunocytochemistry

Cells were grown on a six-well cell culture slide, fixed with 4% formaldehyde (Sigma-Aldrich) at 4 °C for 30 min, permeabilized with 0.1% Triton X-100 in phosphate-buffered saline (PBS), and blocked with 10% normal goat serum (Invitrogen) or 3% bovine serum albumin (Sigma-Aldrich) for 1 h at room temperature. Subsequently, antibodies against Oct3/4 (SC-5279, Santa Cruz Biotechnology), Nanog (NBP2-19469, Novus Biological), MAP2 (AB5622, Millipore), Tubb3 (MAB1637, Millipore), pERK1/2 (4370, Cell Signaling), Hif2α (NB100-122, Novus Biological), Nox4 (PA1-46014, Thermo Fisher Scientific), and Vps26a (AB23892, Abcam) were incubated with the prepared cells at 4 °C overnight. Finally, the cells were washed several times with 0.02% Tween-20 in PBS (PBST) and incubated with Alexa Fluor 488 or Alexa Fluor 594 secondary antibodies (Invitrogen). Fluorescence was analyzed by fluorescence microscopy (Leica). Quantification of the fluorescence intensity or positive cells/nuclear ratios was performed using ImageJ software. Representative mean values were of at least three independent experiments with standard deviation.

### Plasmid construction

The pcDNA6/Myc-His-Vps26a plasmid was constructed as follows. Full-length mouse Vps26a cDNA was amplified by PrimeSTAR^®^ HS DNA polymerase (TaKaRa) using mouse ESC cDNA as the template. The Vps26a-Myc-His-expressing plasmid was generated by ligating the resulting DNA into the pcDNA6/Myc-His vector (Invitrogen) following digestion with EcoRI and XhoI. The integrity of the Vps26a cDNA was confirmed by sequencing. The expression of Vps26a was analyzed by immunoblotting using specific Myc (TA150081, Origene) or Vps26a (AB23892, Abcam) antibodies. For construction of the C-terminally GST-tagged Vps26a, the cDNA encoding GST and Vps26a WT (1–360 aa), or deletion constructs containing amino acids 1–148, 149–245, or 246–360, were amplified and inserted into the pEBG-GST mammalian expression vector (Addgene #22227). The HA-tagged Nox4C (C-terminal region, 249–574 aa) expression plasmids were kindly provided by Yun-Soo Bae (Ewha Woman's University, Seoul, Korea).

### Stable transfection of cells by lentiviral vector-mediated gene knockdown

For the shRNA-mediated knockdown of *Vps26a*, pLKO.1 TRC library clones against *Vps26a* were used (Sigma-Aldrich, clone TRCN0000366609; 5′-AGG UGU UCA GCC AGA CCA C-3′), and a scramble non-silencing shRNA served as the control [pLKO.1-puro non-mammalian shRNA control (Sigma-Aldrich, SHC002V; 5′-CAA CAA GAT GAA GAG CAC CAA-3′)]. P19 ECCs were stably transfected with the lentiviral vectors pLKO.1-puro-CMV-Vps26a or pLKO.1-puro-shRNA Control. Stable cell lines were obtained after selection by long-term culture in medium containing 2 μg/ml puromycin (Invitrogen) for 20 days. Colonies were manually picked and further expanded in the presence of puromycin to establish individual knockdown lines. Each line was evaluated by RT-PCR and western blotting to confirm efficient knockdown of Vps26a.

### Chemical treatment

To induce neurogenesis, ESCs were treated from day 0 to 6 with NBM (DMEM/F12 1:1 with N2 supplement and B27 supplement, GIBCO) and P19 ECCs using two differentiation methods: (1) cultured from day 0 to 6 with NBM and (2) treated from day 0 to 2 with NBM containing 0.5 μM RA (Sigma-Aldrich). For chemical treatment during neurogenesis, ESCs and P19 ECCs cells were treated with 50 and 100 μM hydrogen peroxide (H_2_O_2_; Sigma-Aldrich), 2.5 mM NAC (Sigma-Aldrich), and 10 μM DPI (Sigma-Aldrich) for the indicated times, and MAPK inhibitors (all from Calbiochem), such as p38 MAPK (5 μM SB203580), ERK1/2 (10 μM PD98059 or 10 μM U0126), PI3K (10 μM LY294002), and JNK (5 μM SP600125) were added at 12 h intervals. HEK293 cells were treated with 10 ng/ml epidermal growth factor (EGF, ERK 1/2 signaling activator; Sigma-Aldrich).

### Measurement of intracellular ROS

Intracellular ROS generation was assessed using the ROS indicator 5,6-chloromethyl-2′,7′-dichlorodihydrofluorescein diacetate (CM-H_2_DCFDA; Invitrogen). Exponentially growing cells were incubated with 5 μM CM-H_2_DCFDA at 37 °C for 30 min, washed twice with PBS, and centrifuged at 200 × *g* for 5 min. The DCFDA fluorescence intensity was measured immediately by flow cytometry with a FACSCalibur instrument (BD Biosciences) or by fluorescence microscopy (Zeiss Axiovert 200 M).

### Immunoprecipitation and GST pull-down assays

Immunoprecipitations were performed as described previously [48]. Briefly, protein samples from ~ 10^7^ cells were incubated with 3 μg of specific antibody overnight at 4 °C. The resulting immunocomplexes were then incubated with protein G-conjugated magnetic beads (DYNAL, Invitrogen) for 2 h at 4 °C. The samples were washed four times with a digestion buffer supplemented with 0.1% NP-40 (protein lysis buffer) at room temperature. The proteins were then eluted by incubation with 0.4 M NaCl TE buffer for 30 min and analyzed by western blotting. GST pull-down assays were performed as previously described [49], with some modifications. Solubilized striatal extracts containing 50–100 μg protein were diluted with 1 × PBS/1% Triton X-100, incubated with a 50% (v/v) slurry of glutathione-Sepharose 4B beads (GE Healthcare), and saturated with GST alone or GST-fusion protein (5–10 μg) at 4 °C for 2 h. The beads were then washed four times with 1× PBS/1% Triton X-100. All bound proteins were eluted with 4× lithium dodecyl sulfate loading buffer, resolved by SDS-PAGE, and immunoblotted with specific antibodies.

### Teratoma assays

Approximately 10^7^ cells were injected subcutaneously into the hind leg of BALB/c nude mice. Teratomas were recovered when tumor formation became outwardly apparent. They were fixed in Bouin’s fixative for hematoxylin and eosin staining or with paraformaldehyde for immunohistochemistry.

### Immunohistochemistry

Immunohistochemistry was performed as described previously [19]. Briefly, teratomas were fixed overnight with 10% neutral-buffered formalin, embedded in paraffin, and processed into 5-mm thick sections. For antigen retrieval, deparaffinized sections were heated for 4 min in a pressure cooker containing 10 mM citrate buffer (pH 6.0). Subsequent procedures were performed at room temperature. Sections were pretreated with 3% H_2_O_2_ in 0.1 M TBS for 30 min to quench endogenous peroxidases and then treated with a protein blocking solution (Dako) for 20 min. Next, they were incubated with antibodies against Oct3/4 (SC-5279, Santa Cruz Biotechnology), Tubb3 (MAB1637, Millipore), Nox4 (14347-1-AP, Proteintech), and Vps26a (AB23892, Abcam) for 30 min in a humidified chamber. The sections were washed with TBST followed by incubation with EnVision anti-rabbit polymer (Dako) for 30 min. The peroxidase bound to the antibody complex was visualized by treatment with 3,30-diaminobenzidine chromogen substrate solution (Dako). The color reaction was monitored under a microscope to determine the optimal incubation time, and the reaction was stopped by several washes with TBS. Immunolabeled sections were dehydrated in a graded ethanol series, defatted in xylene, mounted, and examined under a bright-field using an Olympus BX51 microscope (Olympus). Images were acquired using an Olympus DP 70 camera.

### Statistical analyses

The data are the means and standard deviation (SD) from three independent experiments. Differences among groups were calculated for significance (*P* < 0.05) using Prism software. Experimental differences between two groups were calculated via the two-tailed *t* test, and differences among three or more groups were calculated via one-way analysis of variance and the Bonferroni post-hoc test. *P* < 0.05 was considered to indicate statistical significance and is indicated on graphs by an asterisk. *P* < 0.01 and < 0.001 are indicated by two and three asterisks, respectively.

## Electronic supplementary material

Supplementary Information
