# Supplementary material for: Novel crosstalk between Vps26a and Nox4 signaling during neurogenesis
Source: Cell Death Differ. 2018 Nov 21;26(9):1582–99. doi: 10.1038/s41418-018-0226-0 (PMC6748115; doi:10.1038/s41418-018-0226-0)
Supplement: Supplementary file 1 — Supplementary Information [file 41418_2018_226_MOESM1_ESM.pdf]

SUPPLEMENTAL MATERIALS

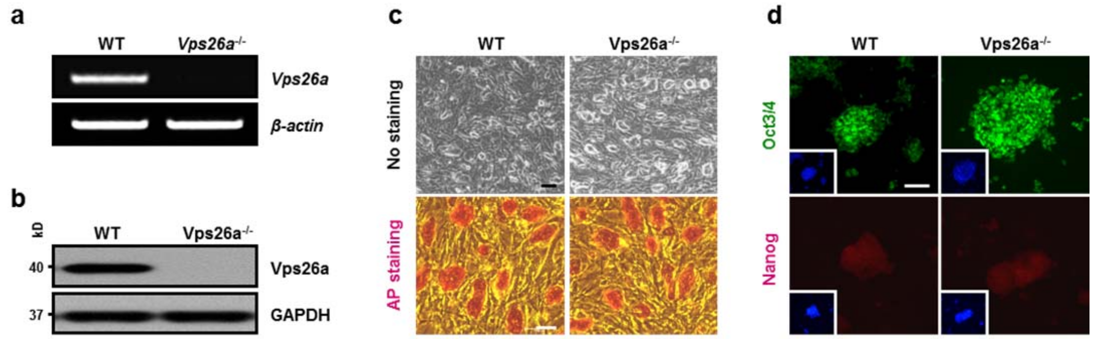

Supplementary **Figure 1** Derivation of ESCs from Vps26a-null blastocysts. **(a, b)** Semi-qPCR and Western blot analyses of Vps26a using WT and *Vps26a*$^{-/-}$ ESCs. **(c)** Photographs of exponentially growing WT and *Vps26a*$^{-/-}$ ESCs and AP activity assay. Scale bars, 200 μm (upper images) and 50 μm (bottom images). **(d)** Immunocytochemical analysis of Oct3/4 (green) and Nanog (red) using WT and *Vps26a*$^{-/-}$ESCs. DAPI staining data are shown as insets to the respective images. Scale bar, 50 μm.

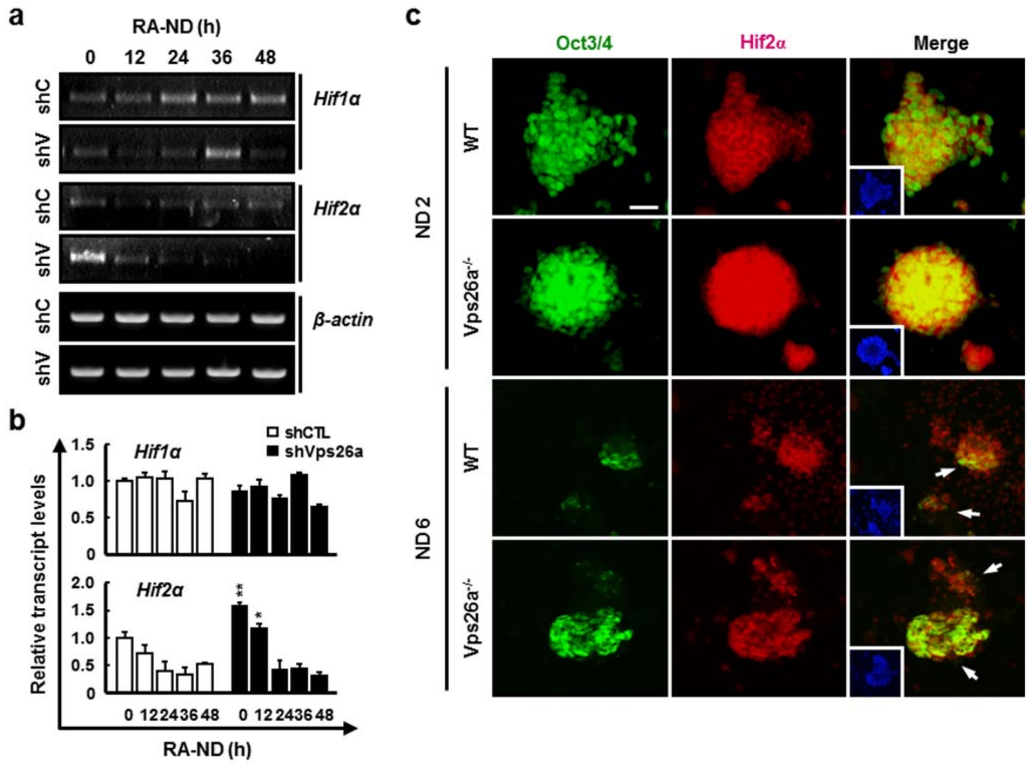

Supplementary **Figure 2** Vps26a deficiency increases HIF2α during ESC-mediated neurogenesis. **(a,b)** The effect of Vps26a deficiency (a) or knockdown (b) on expression of *Hif1α* and *Hif2α* determined by semi-qPCR and qPCR analyses using ECCs, respectively, differentiated for the indicated time periods. Error bars indicate the means ± SD (n = 3). $^{*}P < 0.05$; $^{**}P < 0.01$ compared to shCTL-ECCs for each day during RA-ND. **(c)** Double-label immunocytochemical analysis of Hif2α (red) and Oct3/4 (green) using WT and *Vps26a*$^{-/-}$ ESCs differentiated for 2- (upper panel) and 6 days (bottom panel). DAPI staining data are shown as insets to the merged images (yellow). Scale bar, 50 µm.

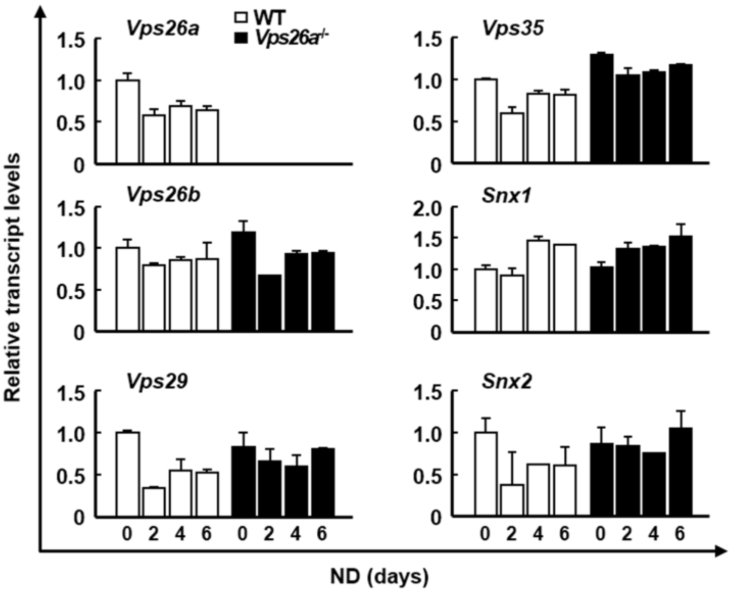

Supplementary **Figure 3** Expression profiles of the retromer complex genes during neurogenesis from ESCs. The effect of *Vps26a*-deficiency on the expression of retromer complex genes (*Vps26a, Vps26b, Vps29, Vps35, Snx1* and *Snx2*) determined by semi-qPCR analysis using ESCs differentiated for the indicated time periods.

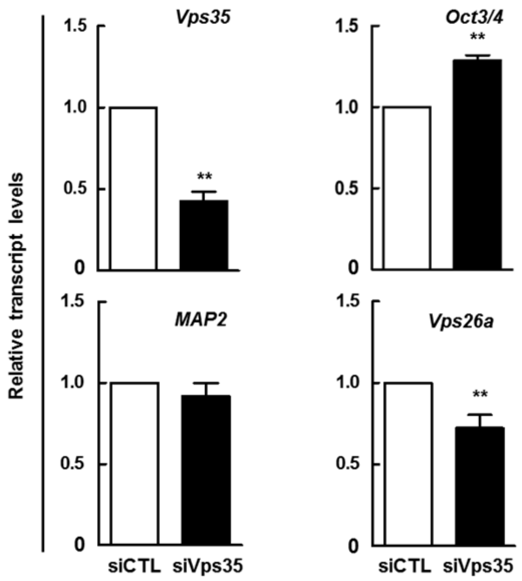

Supplementary **Figure 4** Knockdown effect of Vps35 in RA-ND. The effects of Vps35 on the expression of Oct3/4, MAP2 and Vps26a were determined by qPCR using siCTL- and siVps35-ECCs in RA-ND. Error bars indicate the means ± SD (n = 3). $^{*}P < 0.05$; $^{**}P < 0.01$ compared to the siCTL RA-ND 48h.

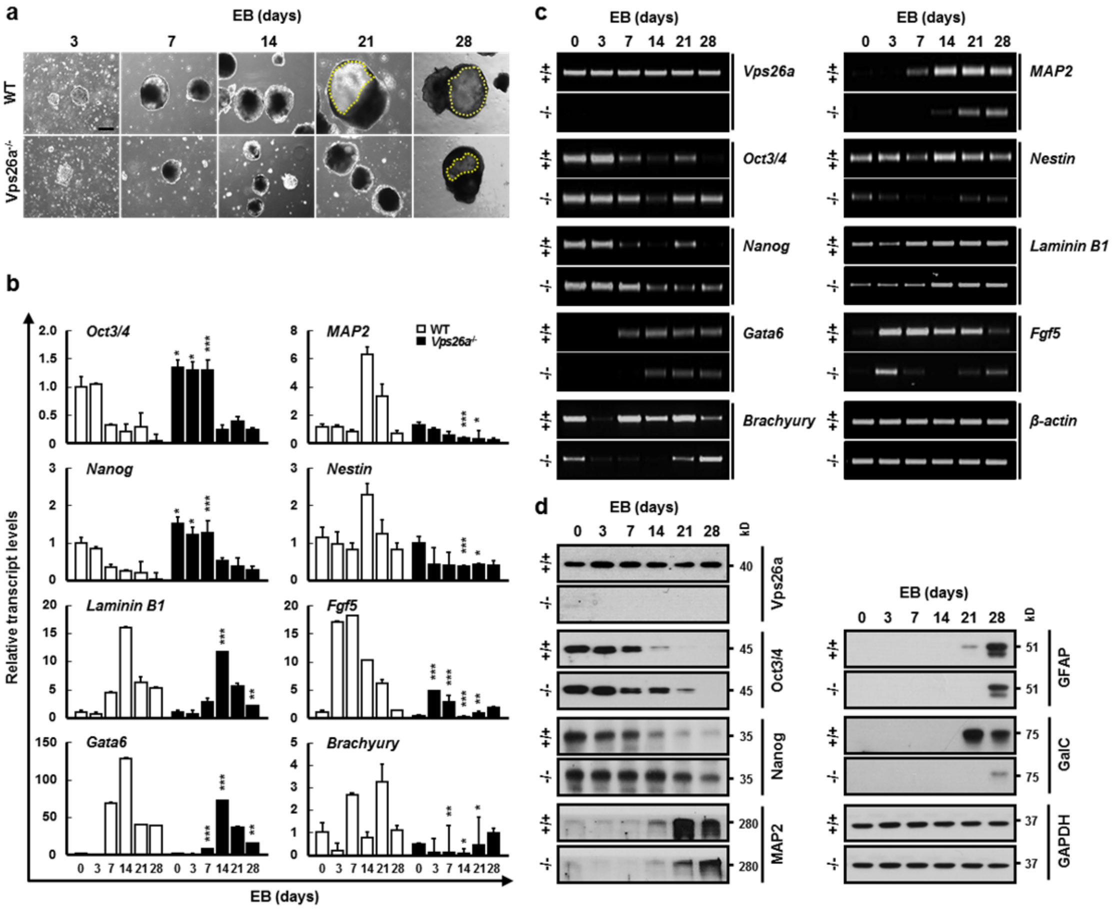

Supplementary **Figure 5** Vps26a deficiency fails to activate differentiation in three germ layers during EB formation**. (a)** Representative images of WT and *Vps26a*$^{-/-}$ EBs formed over the indicated culture periods. Dotted yellow lines indicate cystic cavities. Scale bar, 50 µm. **(b, c)** Expression kinetics of ESC stemness and lineage-specific markers, including ectoderm (*MAP2*, *Nestin* and *Fgf5*), mesoderm (*Brachyury*) and endoderm (*Gata6* and *Lanimin B1*) were measured by semi-qPCR (b) and qPCR (c) analyses using WT (+/+) and *Vps26a*$^{-/-}$ (-/-) EBs formed over the indicated culture periods. Error bars indicate the means ± SD (n = 3). $^{*}P < 0.05$; $^{**}P < 0.01$; $^{***}P < 0.001$ compared to the WT cells each day during EBs. **(d)** Western blot analysis for ESC stemness and neural differentiation markers (neuron, MAP2; astrocyte, GFAP; oligodendrocyte, GalC) using WT (+/+) and *Vps26a*$^{-/-}$ (-/-) EBs formed over the indicated culture periods.

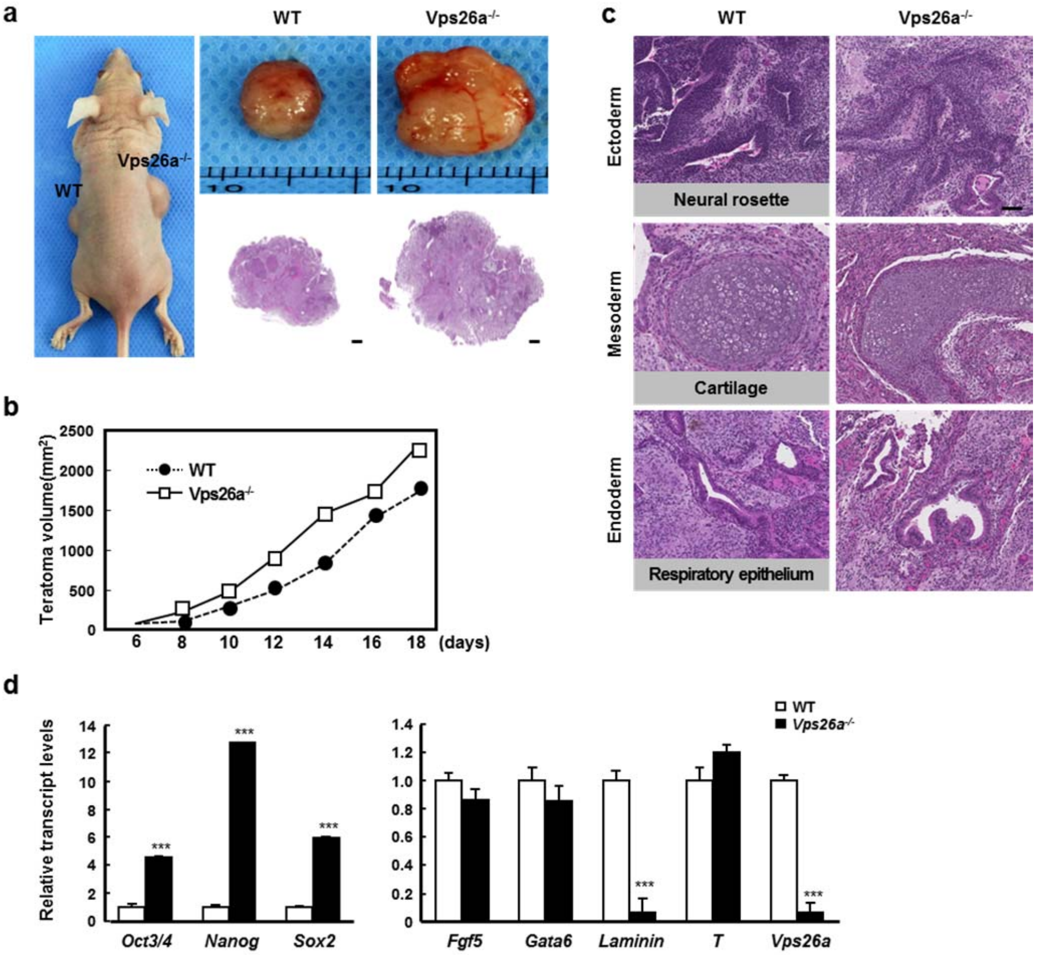

Supplementary **Figure 6** The *in vivo* differentiation of WT and *Vps26a*$^{-/-}$ ESCs into all three germ layers. **(a)** Teratoma formation and histological sections of teratomas formed 2–3 weeks after the subcutaneous injection of WT and *Vps26a*$^{-/-}$ ESCs into BALB/c nude mice. Scale bar, 500 µm. **(b)** Various tissues in the three germ layers were analyzed using hematoxylin and eosin staining. Scale bar, 50 µm. **(c)** The change in teratoma size over time was determined by measuring the length and width at the indicated times for 18 days. **(d)** Expression kinetics of ESC stemness and lineage-specific markers were measured by qPCR (C) analyses using WT and *Vps26a*$^{-/-}$ teratomas. Error bars indicate the means ± SD (n = 5). $^{***}P < 0.001$ compared to the WT teratomas.

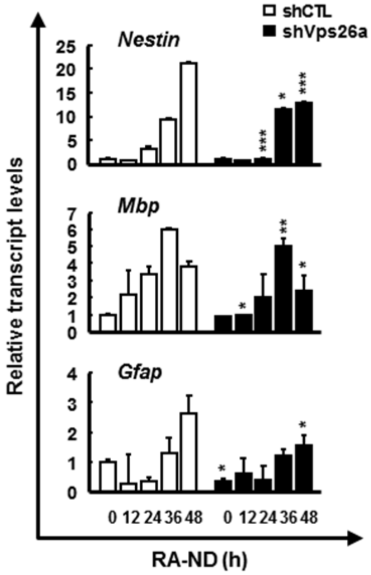

Supplementary **Figure 7** The effect of Vps26a knockdown on glial differentiation from P19 ECCs. shCTL- and shVps26a-ECCs were differentiated in RA-NBM for 48 h and subjected to qPCR analysis of *Nestin*, *Mbp* and *Gfap*. Error bars indicate the means ± SD (n = 3). $^{*}P < 0.05$; $^{**}P < 0.01$; $^{***}P < 0.001$ compared to shCTL for each day during ND.

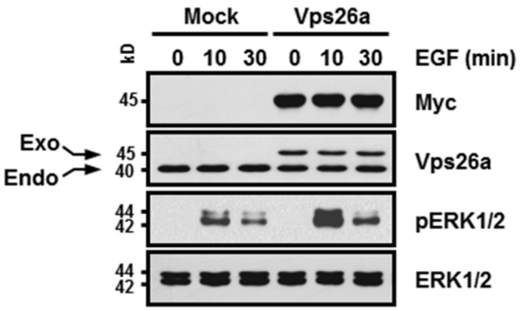

Supplementary **Figure 8** The effect of Vps26a overexpression on ERK1/2 activation in HEK293 cells. HEK293 cells transiently transfected with the vector overexpressing Vps26a (pcDNA6/Myc-hVps26a) were cultured under serum-free condition for 6 h, stimulated with 10 ng/ml of EGF for the indicated periods, and subjected to Western blot analysis of Myc, Vps26a, pERK1/2 and ERK1/2.

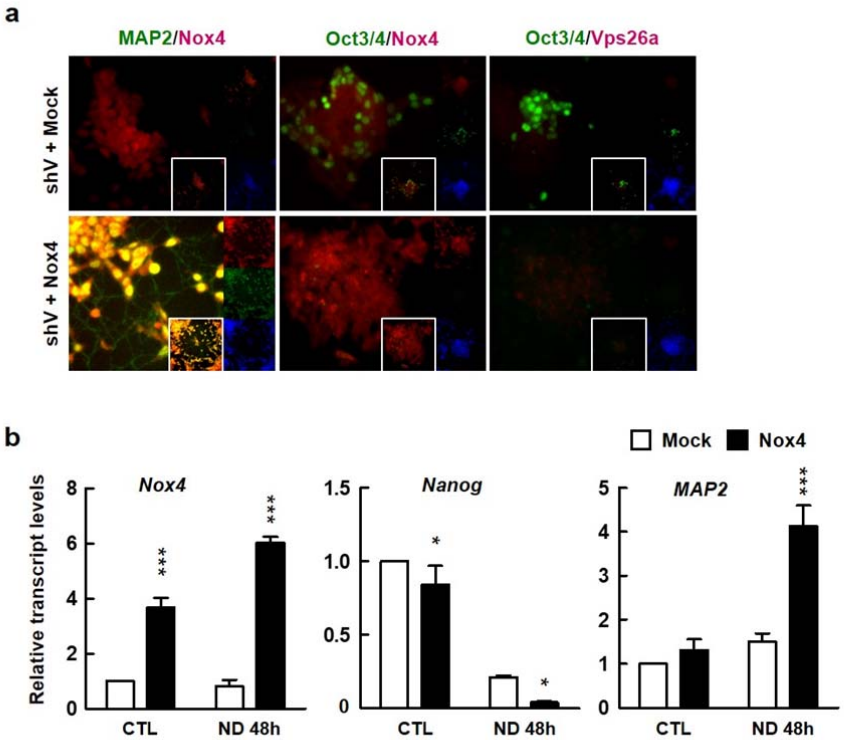

Supplementary **Figure 9** The effect of Nox4 overexpression on RA-ND from shVps26a-ECCs. shVps26a (shV) -ECCs transiently transfected with the vector overexpressing Nox4 (pCMV6-Myc-DDK-Nox4) or Mock were differentiated in RA-NBM for 48 h. (a) Double-label immunocytochemical analysis of MAP2 (green), Oct3/4 (green), Nox4 (red) and Vps26a (red). DAPI staining data are shown as insets to the merged image. Scale bar, 50 μm. (b) The mRNA level of *Nox4*, *Nanog* and *MAP2* were evaluated via qPCR analysis. Error bars indicate the means ± SD ($n = 3$). $^{*}P < 0.05$; $^{***}P < 0.001$ compared to the shVps26a+Mock.

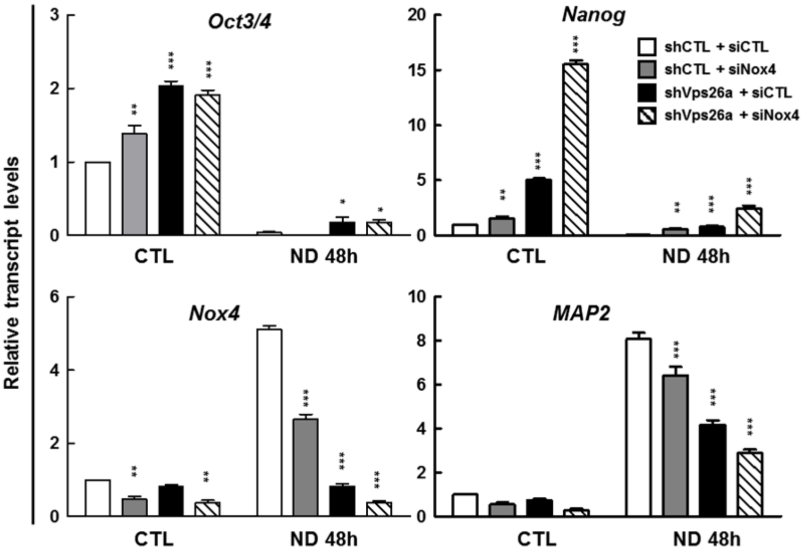

Supplementary **Figure 10** Decreased expression of Nox4 restores stemness and inhibits neurogenesis. After shCTL and shVps26a-ECC were transfected with siCTL and siNox4, qPCR analysis was performed on *Oct3/4*, *Nanog*, *Nox4* and *MAP2*. Error bars indicate the means ± SD (n = 3). $^{*}P < 0.05$; $^{**}P < 0.01$; $^{***}P < 0.001$ compared to the siCTL.

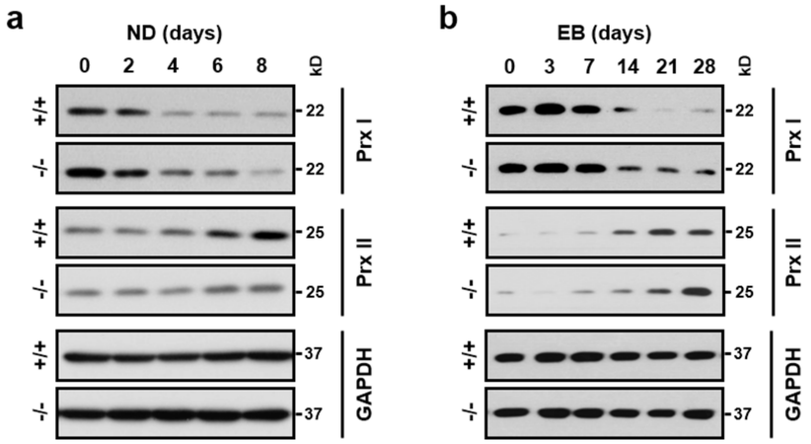

Supplementary **Figure 11** Expression profiles of Prx I and Prx II during ESC-mediated neurogenesis. **(a, b)** The effect of *Vps26a* deficiency on expression of Prx I and Prx II determined by Western blot analysis using ESCs (a) and EBs (b) differentiated for the indicated time periods. GAPDH was used as the loading control. +/+, WT; -/-, *Vps26a*$^{-/-}$.

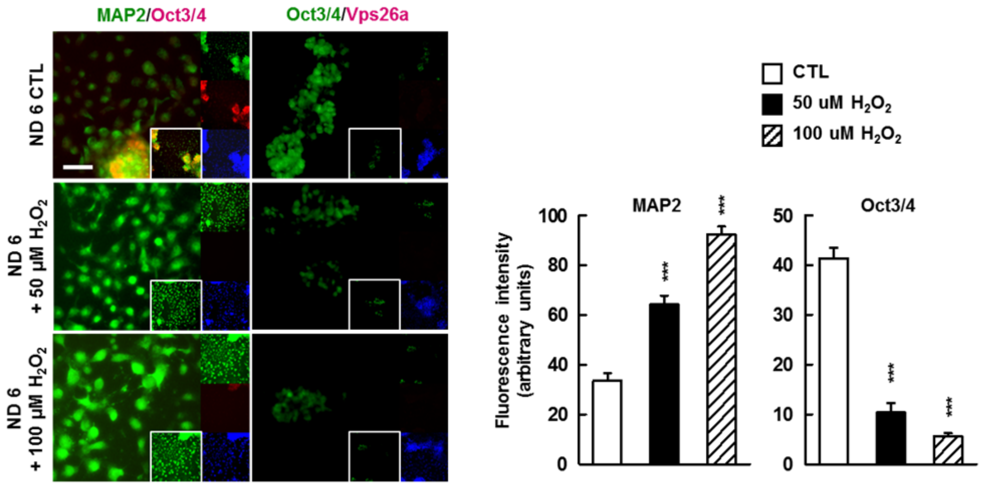

Supplementary **Figure 12** Effects of H_2O_2 on the expression of Oct3/4 and MAP2 during neural differentiation of *Vps26a*$^{-/-}$ ESCs. *Vps26a*$^{-/-}$ ESCs were differentiated in the presence or absence of H_2O_2 (50 μM and 100 μM) for 4 days and subjected to immunocytochemical analysis of MAP2 and Oct3/4. Quantitative analysis of the fluorescence intensity was analysis by Image J software (n = 3 for each group). Error bars indicate the means ± SD. $^{***}P < 0.001$ compare to the *Vps26a*$^{-/-}$ ND 6 CTL.

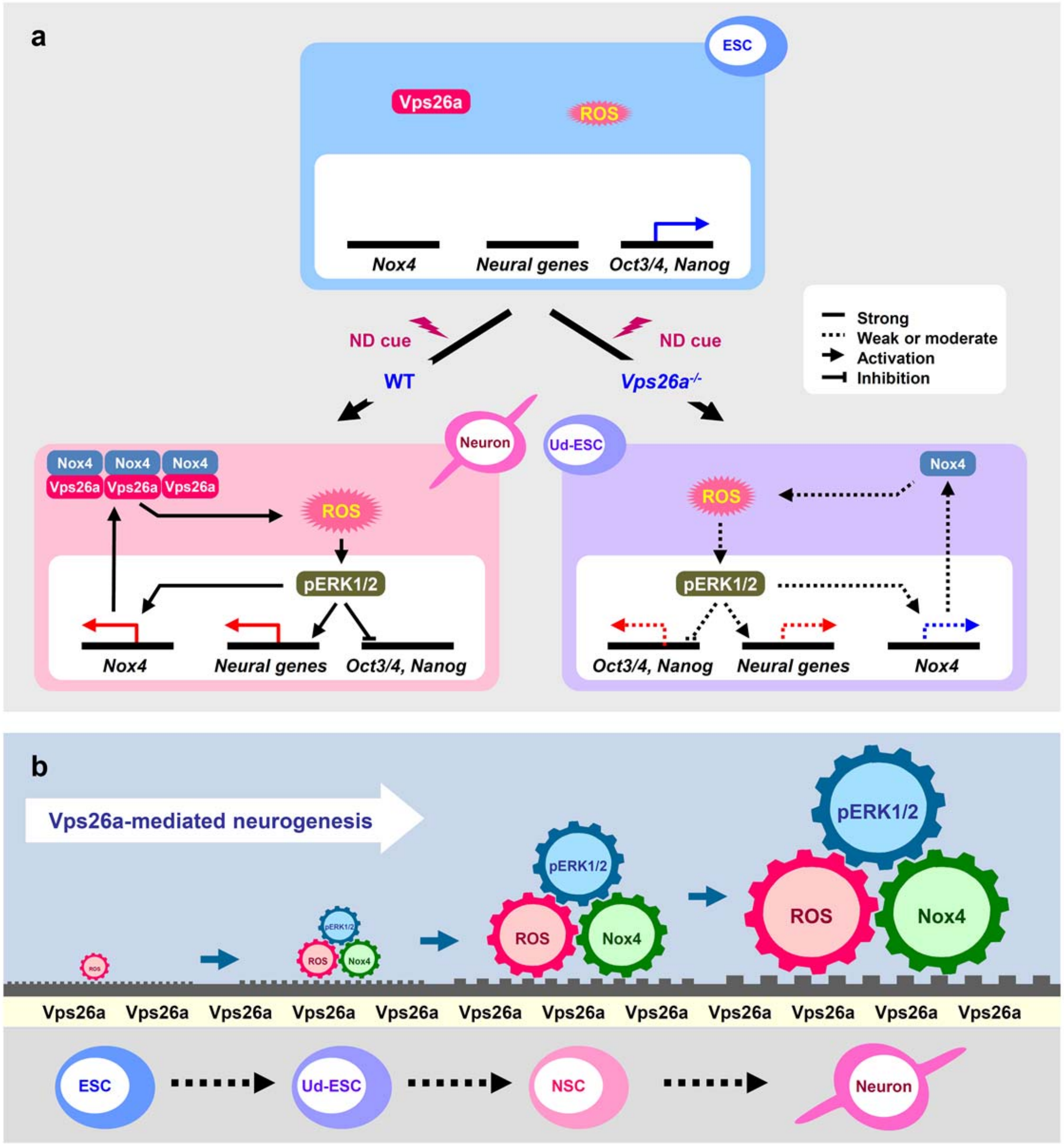

Supplementary **Figure 13** Schematic illustration of a hypothetical model for ESC-mediated neurogenesis governed by interaction between Vps26a and Nox4 promote activation of the ROS/ERK1/2 axis. **(a)** In self-renewing ESCs, ROS are maintained at lower levels. Under a differentiation stimulus, Nox4 expression was upregulated, causing a significant increase in ROS levels, and leading to activation of the ERK1/2 signaling pathway. The activated ERK1/2 shut down

the expression of stemness markers and induced transcription of neural genes. In addition, Vps26a interacted with Nox4 was further upregulated by the activated ERK1/2 pathway, which resulted in a large increase in ROS levels and subsequent hyper-activation of ERK1/2. Ultimately, many more neural genes were switched on by the activated Nox4/ROS/ERK1/2 cascade, and ESCs actively differentiated into neurons. However, the cascading between Nox4, ROS and ERK1/2 was severely impaired in differentiating *Vps26a*$^{-/-}$ ESCs, enabling the ESCs to maintain their undifferentiated state (Ud-ESCs) for a relatively long period compared to the WT. **(b)** Crosstalk between Vps26a and the Nox4/ROS/ERK1/2 cascade leads to ESC-mediated neurogenesis. Vps26a-mediated stemness/differentiation transition was highly dependent on the cyclic cascade between Nox4, ROS and ERK1/2.

Supplementary Table 1. Information on PCR primers used in this study.

**Target genes**	**Accession number**	**Primer sequences**		**Product size (bp)**
		**Forward**	**Reverse**	
Oct3/4	NM_013633.3	ggtggaaccaactcccgagg	acctttccaaagagaacgccc	150
Nanog	NM_028016.3	caaaggatgaagtgcaagcgg	ggtgctgagcccttctgaatc	80
MAP2	NM_008632.2	actgccggacctgaagaatg	agtaacaatttgtacctgacccc	91
Tubb3	NM_023279.2	atgtcgtgcggaaagagtgt	cttgctgatgagcagtgtgc	110
Nestin	NM_016701.3	catacaggactctgctggagg	aggtgctggtcctctggtat	130
Mbp	NM_001025256.2	tgtgccacatgtacaaggact	gatggaggtggtgttcgagg	145
Gfap	NM_001131020.1	cagatccgaggggcaaaag	tggcagggctccattttcaa	123
Gata6	NM_010258.3	gctgaacggaacgtaccacc	acagtggcgtctggatggag	236
Laminin B1	NM_008482.2	ccccaatctctgtgaaccatg	gcaatttgcaccgacactga	119
Brachyury	NM_009309.2	ggtggcttgttcctggtgc	gtaggtgggctggcgttat	292
Fgf5	NM_010203.5	aaagtcaatggctcccacgaa	ggcacttgcatggagttttcc	141
Vps26a	NM_133672.3	gaagtgggcattgaagactg	gtgctgggtccaattcctg	170
Vps26b	NM_178027.4	ggacagaatgtgaagctccg	caatcctcaatgccaacttc	152
Vps29	NM_019780.1	cgtccacatcgtgagaggag	tgtcctgagataagaatgtccac	180
Vps35	NM_022997.4	aacacagaaatcgtctctcagg	cagatgaataaatcggccaac	150
Snx1	NM_019727.2	cccttacttctcatcctccg	cataggcattcataccatccc	150
Snx2	NM_026386.1	gatcttttcgcagaagccac	cttcaatctcgtccctggat	184
Hif1α	NM_010431.2	ggcgagaacgagaagaaaaag	gaagtggcaactgatgagca	124
Hif2α	NM_010137.3	gtgacccaagacggtgacat	ctcacggatctcctcatggt	132
Nox1	NM_172203.2	gtaggtgtgcatatgggtgtca	gcctccctaggagcaatctg	105
Nox2	NM_007807.5	ccctccctgtctaggtaatgc	tagcatttgccttcggtgat	102
Nox3	NM_198958.2	gatgtatttcactaccccgtgag	ctcaggcaggctctgtgatt	117
Nox4	NM_015760.5	aacacctctgcctgctcat	acacaatcctaggcccaaca	122
β-Actin	NM_007393.3	cagcttctttgcagctcctt	cacgatggaggggaatacag	157
Gapdh	NM_008084.3	agaacatcatccctgcatcc	cacattgggggtaggaacac	110

Supplementary Table 2. Information of antibodies used in the study.

Antibody	Type	Host	Supplier	Catalog No.
Oct3/4	Monoclonal	Mouse	Santa Cruz Biotechnology	SC-5279
Nanog	Polyclonal	Rabbit	Novus biological	NBP2-19469
MAP2	Polyclonal	Rabbit	EMD Millipore	AB5622
Tubb3	Monoclonal	Mouse	EMD Millipore	MAB1637
GFAP	Monoclonal	Mouse	EMD Millipore	MAB360
GalC	Monoclonal	Mouse	EMD Millipore	MAB342
Vps26a	Polyclonal	Rabbit	Abcam	AB23892
pp38	Polyclonal	Rabbit	Cell Signaling	4511
pAKT	Polyclonal	Rabbit	Cell Signaling	4060
pERK1/2	Polyclonal	Rabbit	Cell Signaling	4370
pJNK	Polyclonal	Rabbit	Cell Signaling	9251
GST	Polyclonal	Rabbit	Cell Signaling	2622
Nox4	Polyclonal	Rabbit	Proteintech	14347-1-AP
Prx I	Polyclonal	Rabbit	Abfrontier	LF-PA0095
Prx II	Polyclonal	Rabbit	Abfrontier	LF-PA0091
HA	Monoclonal	Rat	Roche	11867423001
Myc	Polyclonal	Rabbit	Origene	TA150081
GAPDH	Polyclonal	Rabbit	Abfrontier	LF-PA0212

Supplementary materials and methods

siRNA-mediated knockdown of mRNA

The following small interfering RNA (siRNA) target sequences were used against mouse Vps35 and Nox4. ON-TARGET plus mouse siRNA oligos (Thermo Scientific Dharmacon, Waltham, MA, USA) for Vps35 (L-063309-01-0010) have been previous tested and verified to knockdown Vps35 gene expression[1] and for Nox4 (L-058509-00-0010) have been previous tested and verified to knockdown Nox4 gene expression[2], scrambled siRNA (D-001810-10-20) were transfected into cells using Lipofectamine RNAiMAX Reagent (Life Technologies) according to the manufacturer's instructions.

Nox4 overexpression

For Nox4 overexpression experiments, plasmid DNA encoding mouse Nox4 cDNA (pCMV6-Myc-DDK-tagged; NM_015760, #MR227192; Origene)[3] or empty vector as a control using 4D Nucleofector™ (Lonza), according to the manufacturer's instructions.

Supplementary reference

1. Mir R, Tonelli F, Lis P, Macartney T, Polinski NK, Martinez TN*, et al.* The Parkinson's disease VPS35[D620N] mutation enhances LRRK2-mediated Rab protein phosphorylation in mouse and human. *Biochem J* 2018, **475**(11)**:** 1861-1883.

2. Jayavelu AK, Muller JP, Bauer R, Bohmer SA, Lassig J, Cerny-Reiterer S*, et al.* NOX4-driven ROS formation mediates PTP inactivation and cell transformation in FLT3ITD-positive AML cells. *Leukemia* 2016, **30**(2)**:** 473-483.

3. Moon JS, Nakahira K, Chung KP, DeNicola GM, Koo MJ, Pabon MA*, et al.* NOX4-dependent fatty acid oxidation promotes NLRP3 inflammasome activation in macropha

ges. *Nat Med* 2016, **22**(9)**:** 1002-1012.
